# Blood-triggered generation of platinum nanoparticle functions as an anti-cancer agent

**DOI:** 10.1038/s41467-019-14131-z

**Published:** 2020-01-28

**Authors:** Xin Zeng, Jie Sun, Suping Li, Jiyun Shi, Han Gao, Wei Sun Leong, Yiqi Wu, Minghui Li, Chengxin Liu, Ping Li, Jing Kong, Yi-Zhou Wu, Guangjun Nie, Yuming Fu, Gen Zhang

**Affiliations:** 1 Women’s Hospital of Nanjing Medical University, Nanjing Maternity and Child Health Care Hospital, Nanjing, 210004 China; 2 Department of Cell Biology, School of Basic Medicine, Nanjing Medical University, Nanjing, 211166 China; 3 Safety Assessment and Research Center for Drug, Pesticide and Veterinary Drug of Jiangsu Province, School of Public Health, Nanjing Medical University, Nanjing, 211166 China; 4 CAS Key Laboratory for Biomedical Effects of Nanomaterials & Nanosafety, CAS Center for Excellence in Nanoscience, National Center for Nanoscience and Technology, China, Beijing, 100190 China; 5 Center of Materials Science and Optoelectronics Engineering, University of Chinese Academy of Sciences, Beijing, 100049 China; 6 Key Laboratory of Protein and Peptide Pharmaceuticals, Institute of Biophysics, Chinese Academy of Sciences, Beijing, 100101 China; 7 School of Biological Science and Medical Engineering, Beihang University, Beijing, 100191 China; 8 Department of Electrical Engineering and Computer Science, Massachusetts Institute of Technology, Cambridge, MA 02139 USA; 9 Australian Institute for Bioengineering and Nanotechnology, The University of Queensland, Brisbane Qld, 4072 Australia; 10 Beijing Advanced Innovation Center for Biomedical Engineering, Beihang University, Beijing, 100083 China

**Keywords:** Drug delivery, Cancer therapy, Medical research, Nanoparticles

## Abstract

Since the discovery of metal nanoparticles (NPs) in the 1960s, unknown toxicity, cost and the ethical hurdles of research in humans have hindered the translation of these NPs to clinical use. In this work, we demonstrate that Pt NPs with protein coronas are generated in vivo in human blood when a patient is treated with cisplatin. These self-assembled Pt NPs form rapidly, accumulate in tumors, and remain in the body for an extended period of time. Additionally, the Pt NPs are safe for use in humans and can act as anti-cancer agents to inhibit chemotherapy-resistant tumor growth by consuming intracellular glutathione and activating apoptosis. The tumor inhibitory activity is greatly amplified when the Pt NPs are loaded in vitro with the chemotherapeutic drug, daunorubicin, and the formulation is effective even in daunorubicin-resistant models. These in vivo-generated metal NPs represent a biocompatible drug delivery platform for chemotherapy resistant tumor treatment.

## Abstract (web summary)

Platinum based drugs like cisplatin are common chemotherapy treatments for cancer. Here, the authors report on the in situ formation of platinum nanoparticles in patients and demonstrated how platinum nanoparticles can be synthesized using patients’ blood and provide effective drug delivery and cancer treatments.

## Introduction

In the burgeoning field of nanotechnology, there has been an upsurgein the use of metals or metal compound nanoparticles (NPs) in living organisms. For instance, biosynthesis of CdTe quantum dots in earthworms generates metal NPs that are useful for live cell imaging^1^. Upon the discovery of magnetite particles in the human brain, iron oxide NP studies have revealed unique combinations of redox activity, surface charge and magnetic behavior^2^. The presence of iron oxide NPs in the brain insinuates that metal NPs can be safely applied in the human body. Indeed, several agents containing heavy metals are clinically administered. For example, platinum (Pt) is the main component in the anti-cancer drug cisplatin^3^, a mercury adjuvant is used in traditional Chinese medicine^4^ and gadolinium-containing contrast agents are used for radiology^5^. Since cisplatin is approved as a first line treatment for ovarian, testicular, lung and bladder cancers, as well as lymphomas, myelomas and leukemias, various cisplatin-based complexes have been generated for additional applications in cancer treatment^6^.

Based on the physicochemical properties of metals, the physiological milieu favors their transformation into metal NPs. We have previously demonstrated that ZnO NPs (bio-ZnO NPs) are spontaneously biosynthesized when animals are fed Zn ions in aqueous solution^7^. In addition, NPs generally become coated with a protein corona in the circulation. For example, Pt NPs become naturally coated with albumin to realize the same tumor-targeting properties as the FDA-approved paclitaxel-albumin complexes^8^. Interestingly, noble metal-based NPs (e.g., Pt, Au, Ag) can spontaneously absorb GSH, with the sulfhydryl group as a capping ligand^9^. This feature is important to cancer therapy since an increase in reduced glutathione (GSH) activity is the major mechanism of multidrug resistance in carcinomas^10^.

Here, we report the detection of biologically-formed Pt NPs in the circulation immediately after chemotherapy in humans. The size of these Pt NPs permits a long circulation half-life with a slow permeation through the glomerular filtration barrier. The dual tumor-targeting NPs accumulate in tumor regions, exert a prolonged effect via controlled drug release, exhibit good biocompatibility and reverse tumor cell drug resistance. Compared with other heavy metal elements (e.g., Ag, Cd) that are commonly used in the laboratory synthesis of NPs, Pt NPs that are autologously synthesized in a patient blood can be directly applied to cancer treatment without any modification.

## Results

### Characterization of Pt NPs in human blood

**Fig. 1:**
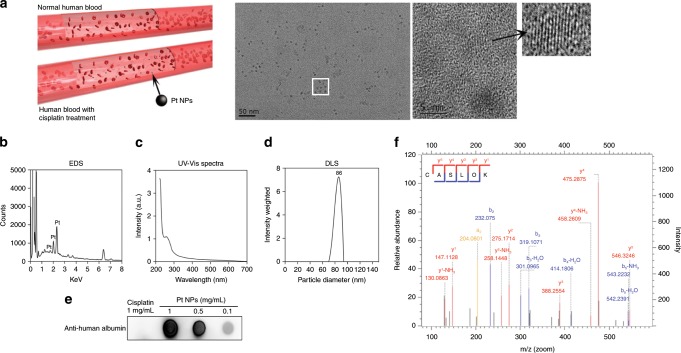
Characterization of Pt NPs. **a** Illustration of the biosynthesis of the Pt NPs in human blood. TEM image of Pt NPs and high resolution-TEM image of the lattice fringes. **b** EDS spectrum of Pt NPs isolated from patient blood. **c** UV−vis absorption spectrum of HPLC-purified Pt NPs from patient blood. **d** The size distribution of Pt NPs in phosphate buffer was characterized by DLS. **e** Dot blot assay to detect human serum albumin in Pt NPs. Cisplatin was used as the control. **f** Identification of albumin in Pt NPs by MS. The MS spectra and the resulting peaks were searched against the Swiss-Prot protein sequence database. The unique peptide sequence identified was CASLQK (Cys-Ala-Ser-Leu-Gln-Lys).

On a standard chemotherapy regimen, the adult cisplatin dosage is 90 mg m^−2^ ^11^. When the patients in our study were treated with cisplatin by intravenous injection (Supplementary Table 1), Pt NPs were biosynthesized in the blood. To examine the Pt NPs in human blood after chemotherapy, 2 mL blood was collected 24 h post injection (P.I.) and isolated by differential centrifugation, varying from10^3^ to 10^5^ × *g*, at 4 °C. Transmission electron microscopy (TEM) analysis revealed the presence of Pt NPs with an average diameter of 6–8 nm and clear lattice fringes of 0.225 nm (Fig. 1a), which is consistent with the previously reported interplanar spacing of Pt NPs^12,13^. Energy dispersive X-ray spectroscopy (EDS) confirmed the presence of Pt (Fig. 1b). We purified the Pt NPs by high performance liquid chromatography (HPLC) and found them to have a UV-visible spectroscopy absorption peak at about 265 nm (Fig. 1c). By electron microscopy (120 kV) of biological samples containing the Pt NPs (Supplementary Fig. 1, 20–30 nm), the average particle size of the Pt NPs was approximately 86 nm in phosphate buffer (PBS) (Fig. 1d). The zeta potentials of Pt NPs are about −9 mV in both PBS and fetal bovine serum (FBS), showing that the Pt NPs possess colloidal stability (Supplementary Fig. 2). The clinically used Pt-based chemotherapeutics, such as carboplatin, oxaliplatin and nedaplatin, can all form Pt NPs (Supplementary Fig. 3). We expect that the formation of Pt NPs is the result of the presence of a cisplatin group.

### Mechanism of Pt NP formation in human blood

To understand how Pt NPs form in blood, we sought the substances in the blood that govern the biosynthesis of the NPs. We hypothesized that amino acids, peptides or proteins may act as Pt-reducing agents. Considering albumin is the major component of serum (40 g L^−1^ in blood^14^), serum albumin (SA) seemed a good candidate to play a role in Pt NP synthesis. Therefore, we analyzed the surface residues of HPLC-purified Pt NPs, using a dot blot assay with antibodies against human SA (HSA) to find that Pt NPs are indeed coated with SA (Fig. 1e). We then analyzed the Pt NPs by mass spectrometry (MS). The surface protein fragments were unique fragments of HSA (Fig. 1f). To test if Pt NPs could be synthesized in vitro, we incubated an aqueous solution of SA with cisplatin for 48 h at 37 °C and found that Pt NPs were generated (Supplementary Fig. 4a). Under the same conditions, the amount of Pt NPs was severely decreased when EDTA, which is known to chelate Pt from cisplatin, was added to the reaction (Supplementary Fig. 4b). These results suggest an essential role of SA in the biosynthesis of Pt NPs from cisplatin. Although the coating of Pt NPs in the blood is an intricate process, making it difficult to precisely determine the composition of a NP protein corona^15,16^, the surface proteins on Pt NPs can still be analyzed using current methods. We performed thirteen proteomics experiments, using the PSM (peptide spectrum match) or iBAQ (intensity based absolute quantification) analysis methods to estimate the relative content of the proteins in corona. Next, we quantified the contents of several of the main proteins by ELISA. The mass ratio is 55.48% HSA, 15.91% macroglobulin and 3.02% apolipoprotein (Supplementary Fig. 5). When we administered 1 mg cisplatin to 1 mL human serum, a maximum yield of 10 mg Pt NPs (including 5% Pt and 95% protein) was obtained. The results of EDS-mapping experiments were consistent with those of the MS analysis (Supplementary Fig. 6), confirming that the coating, C-O-N elements corona is composed of protein and the core is Pt NP. Based on the mapping of the C-O-N elements, the thickness of the protein corona on the Pt NPs is about 30 nm. This is considerably larger than the size of the naked Pt NPs, whose average diameter is below 10 nm, but the dynamic light scattering (DLS) of biosynthesized Pt NPs in a hydrated state exhibit a diameter that is around 86 nm (Fig. 1d). The protein corona plays essential roles in maintaining the drug loading and maintaining or conferring tumor-targeting properties of the NP.

Next, we challenged the current understanding that Pt NPs are synthesized inside blood cells^1,17^. We first isolated 2 mL of human peripheral blood cells and cultured them for 48 h in serum-free medium containing cisplatin. No Pt NPs were captured by TEM analysis in the isolated cells (Supplementary Fig. 7a). In contrast, when we incubated serum isolated from human peripheral blood incubated for 48 h with cisplatin, we observed an abundance of Pt NPs under TEM (Supplementary Fig. 7b). These findings demonstrate that the Pt NPs are synthesized in situ in the plasma reactor rather than in the cell reactor.

**Fig. 2:**
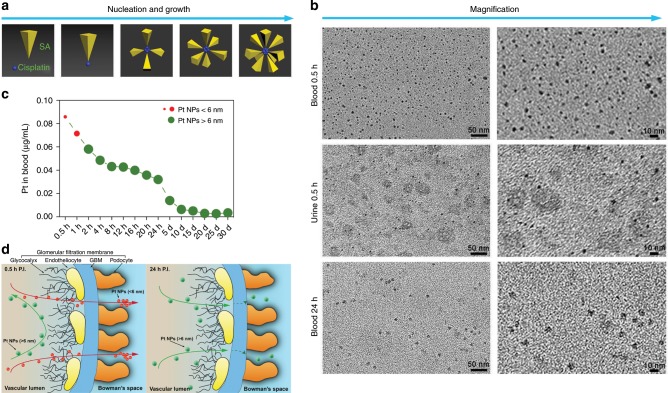
Biosynthesis and metabolism of Pt NPs after cisplatin chemotherapy. **a** Illustration of the process of nucleation and growth of Pt NPs. **b** The varying sizes of Pt NPs in patient blood at 0.5 h after cisplatin treatment (top), in the urine 0.5 h after treatment (middle) and in the blood 24 h after treatment (bottom). The right panels show a higher magnification. **c** The concentration of Pt NPs in patient blood after cisplatin chemotherapy over a 30 d period after treatment. **d** Illustration of the glomerular filtration of different sized Pt NPs; Pt NPs <6 nm more easily traverse the GBM than Pt NPs >6 nm.

There are two stages in the biosynthesis of Pt NPs: nucleation and growth^18^. Based on the clinical pharmacokinetics of cisplatin, there is an initial elimination phase of the drug over the first hour post injection (P.I.), with a circulation half-life of ~15 min^19^. Hence, nucleation and growth are favored and efficiently occurring in the first 0.5 h P.I., due to the highest concentration of cisplatin in the blood (Fig. 2a). Moreover, the zeta potentials of SA and cisplatin are −12.0 and +10.2 mV, respectively. The SA, containing sulfhydryl groups, can form comparatively weak chemical bonds to construct cisplatin-SA complexes (serving as the earliest Pt NP nuclei), which then gradually grow into small Pt NPs in the blood. The 0.5 h interval P.I. is proposed for the initial formation of Pt NPs due to the sufficient supply of the required reactants. Through TEM analysis, we found that Pt NPs in the blood at 0.5 h P.I. appear in a range of sizes (Fig. 2b, top), of which almost 90% are smaller than 6 nm. There were many extremely small Pt NPs; we expect that a number of particles formed at this point were below the TEM resolution (<1 nm).

### Pt NP excretory pathway

We also sought Pt NPs in the chemotherapy patient urine (Supplementary Table 1). At 0.5 h P.I., nearly all urinary Pt NPs were smaller than 6 nm; Pt NPs >6 nm accounted for only about 1% (Fig. 2b, middle panels), suggesting that NPs <6 nm can efficiently filter through glomeruli (Fig. 2d). This is consistent with previous reports, as the pore size of the majority of normal human renal tubular membranes is 2–6 nm, while only about 1% of the pores are larger than 13 nm^20,21^. As time elapsed beyond 0.5 h, the amount of Pt NPs in the urine decreased significantly, especially for those Pt NPs smaller than 6 nm in diameter. At 24 h P.I., we detected few Pt NPs (6–8 nm) in the urine (Supplementary Fig. 8a), however a considerable number of Pt NPs larger than 6 nm were still observed in the blood (Fig. 2b, bottom).

To quantify the concentration of Pt in the blood-derived Pt NPs, we applied inductively coupled plasma–mass spectrometry (ICP-MS) to NPs isolated from patient plasma. The amount of Pt NPs in the blood remained relatively high in the first two hours P.I. (0.058 μg mL^−1^ at 2 h) and gradually decreased with time (0.032 μg mL^−1^ at 24 h; Fig. 2c). This is consistent with an efficient clearance of Pt NPs smaller than 6 nm via the urine. As the cisplatin was cleared or consumed in the blood, de novo Pt NPs became less likely to form, leaving only larger Pt NPs (>6 nm) to grow in vivo. The larger NPs were slowly cleared by the urine in the following days (Fig. 2d). The Pt NPs > 6 nm (0.002 μg mL^−1^) were still present in patient blood at 15 days P.I. and remained detectable even after 30 days (Fig. 2c), suggesting a long circulation half-life in vivo. Thus, the blood behaves as a bioreactor with constant temperature and pressure to biosynthesize metal NPs.

**Fig. 3:**
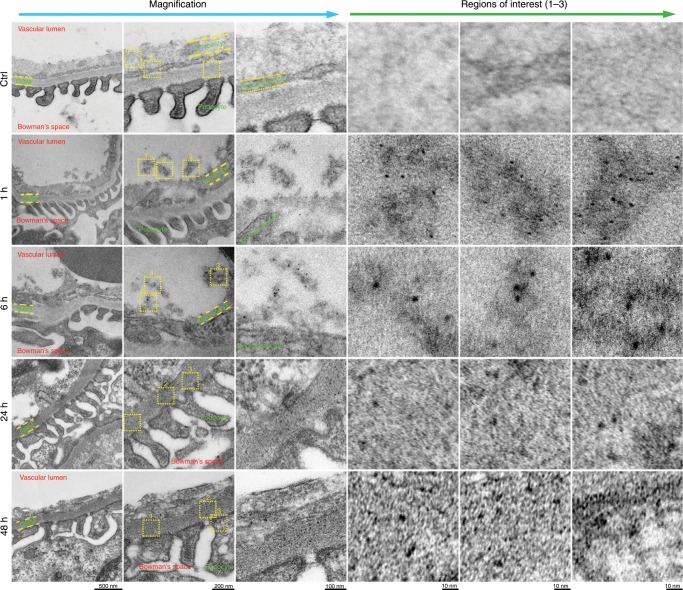
Glomerulus ultrastructure after cisplatin treatment. No NPs were observed in the region of the glomerular filtration membrane, including the glycocalyx, endothelial cells and GBM (*n* = 10), in the control mice treated with PBS (*n* = 10). In the cisplatin-treated mice, a large number of small Pt NPs (<6 nm) were observed at 1 h, while larger Pt NPs (>6 nm) were observed at 6 h. The Pt NPs (>6 nm) were found in the GBM region at 24 h and 48 h, indicating a slow elimination through the glomerulus. Regions of interest are shown at a higher magnification.

To investigate whether Pt NPs undergo glomerular filtration, we analyzed the interaction between Pt NPs and glomeruli using a 120-kV biological TEM (bio-TEM). The glomerulus is the basic filtration unit in the kidney; a tuft of capillaries located within a Bowman capsule^22^. The glomerular endothelium and its glycocalyx, glomerular basement membrane (GBM) and the filtration slits between podocytes perform the basic filtration function of the glomerulus, removing blood borne Pt NPs from the vascular lumen into the Bowman space (Fig. 2d). As shown in Fig. 3, the complete glomerular ultrastructure was apparent, including the endothelial glycocalyx and glomerular endotheliocytes, GBM and podocytes. Although small Pt NPs were observed at 1 h P.I. in the adjacent area of the vascular lumen and endothelial glycocalyx, larger Pt NPs were clearly observable at 6 h P.I. A substantial number of Pt NPs were found in the GBM at 24 h P.I. and in podocytes at 48 h P.I. (Fig. 3), supporting our hypothesis that Pt NPs initiate nucleation and growth in the first 6 h P.I., but can still pass through the GBM and enter the filtrate that forms in the Bowman capsule. Owing to the unique multiple-layer structure of the glomerular filtration barrier, small Pt NPs (<6 nm) can be readily eliminated through the glomerulus. The slower glomerular filtration of large Pt NPs (>6 nm) over the smaller ones is likely caused by a physical retention of the large NPs (>6 nm) in the blood due to spatial steric effects, resulting from the shape and charge of the larger particles (Fig. 2d). For subsequent experiments, we selected Pt NPs with diameters at the critical point for slow filtration (6–8 nm) for drug delivery.

We also examined the biliary excretion pathway. Uptake by the reticuloendothelial system is another common pathway for the human body to remove the larger-sized NPs from the blood, although complete NP removal may require a long time^23^. In the enterohepatic circulation, Pt NPs likely flowed through the liver to the gallbladder, where they would be secreted with the bile into the intestine. We collected the feces from patients treated with cisplatin and detected Pt NPs (>6 nm) at 24 h P.I. (Supplementary Table 1, Fig. 8b). In support of the biliary excretion pathway, we observed Pt NPs (>6 nm) in the hepatic tissue and gallbladder (Supplementary Fig. 8c, d) in mice which were treated intravenously with cisplatin and then exsanguinated. Thus, Pt NPs can be excreted through the enterohepatic circulation, which lacks a membrane barrier, in contrast to glomerular filtration, but the efficiency of this export method is limited due to the slow nature of bile secretion.

### Tumor-targeting and Pt NP distribution in vivo

**Fig. 4:**
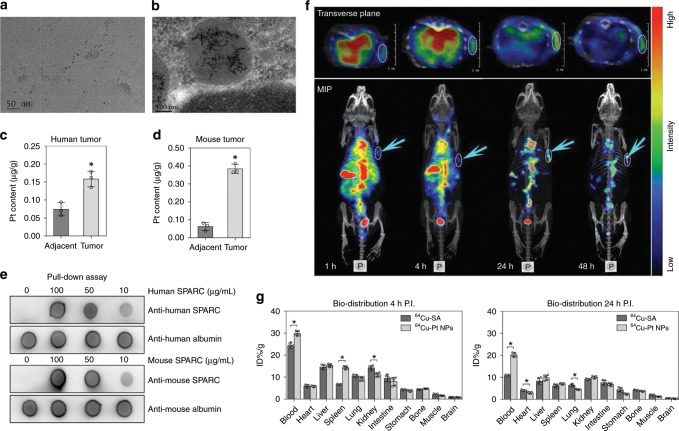
Tumor targeting and bio-distribution of Pt NPs in vivo. **a** Representative TEM image of Pt NPs in a human tumor homogenate. **b** 120 kV bio-TEM showing accumulated Pt NPs in a tumor cell from resected patient tumor tissue. **c** Quantification of the Pt NPs in a human tumor and adjacent, normal tissue (*n* = 3). *P < 0.05. **d** Quantification of the Pt NPs in a mouse tumor and normal, adjacent tissue (*n* = 3). *P < 0.05. **e** *P*ull-down assay to evaluate the interaction of Pt NPs with SPARC. For human-derived Pt NPs, the Pt NPs were incubated with human SPARC proteins at different concentrations, followed by ultra-centrifugal separation. The SPARC-Pt NP complexes were then subjected to dot blot analysis using an anti-human SPARC antibody. An anti-human albumin antibody was applied as the loading control. For mouse-derived Pt NPs, the same experiment was performed, using a mouse-specific SPARC protein and antibody. **f** NanoScan SPECT/CT imaging of ^125^I-Pt NPs in DNR-resistant K562 cell-xenografted nude mice (*n* = 5) at 1, 4, 24 and 48 h after intravenous injection of the NPs. The arrows and dotted circles indicate the tumors. MIP: Maximum Intensity Projection. **g** Time-dependent bio-distribution of ^64^Cu-SA and ^64^Cu-Pt NPs in C57BL/6 mice. The results are presented as the percentage injected dose g^−1^ of tissue (% ID g^−1^). The bars in the histogram represent the results from five independent experiments. *P < 0.05. Data = Mean±Standard deviation.

To better reduce the size or extent of a cancer, neoadjuvant chemoradiotherapy is performed prior to tumor resection. The interval between preoperative chemotherapy and surgery is generally 6–8 weeks^24^. Thus, we collected human tumor tissues after 6 weeks of cisplatin treatment and assessed the presence of Pt NPs (Supplementary Table 2). Tumor samples were obtained from patients who underwent cisplatin chemotherapy and surgery. TEM analyses were conducted to examine the presence of Pt NPs in tumor homogenates (Fig. 4a). Using a 120-kV bio-TEM, we observed that Pt NPs had entered vesicles in the tumor cells (Fig. 4b). We also performed EDS analysis on the Pt NPs in tumor tissues (Supplementary Fig. 9). Quantification of the Pt NP content in tumor tissue by ICP-MS revealed a tumor Pt NP uptake of 0.158 ± 0.023 μg g^−1^, which is two-fold greater than that of the adjacent, normal tissue (0.073 ± 0.018 μg g^−1^), indicating that the Pt NPs target the tumor sites (Fig. 4c). Selective accumulation of Pt NPs is attributed to the enhanced permeability and retention (EPR) effect, which arises from leaky vasculature and minimal lymphatic drainage of tumors, leading to retention of nanosized particles. ICP-MS experiments revealed that the concentration of Pt NPs in tumors remained high, even 11 weeks after the cisplatin injection, indicating Pt NPs aggregate in tumor regions over a longtime period (Supplementary Fig. 10a).

Daunorubicin (DNR), a toxic, anthracycline, aminoglycoside antineoplastic, has been approved by the FDA for the treatment of leukemia and other neoplasms. We examined the tumor-targeting ability of Pt NPs by establishing a DNR-resistant K562 leukemia-xenografted nude mouse model. After a 1.00 μg g^−1^ intravenous injection of cisplatin, the presence of Pt NPs in the tumors was confirmed by TEM analysis (Supplementary Fig. 10b). We further quantified the amount of Pt NPs in tissues at 12 h P.I. and found 0.388 ± 0.018 µg g^−1^ in the tumor, which contained a six-fold greater amount of Pt NPs than the adjacent, normal tissue (0.061 ± 0.023 µg g^−1^; Fig. 4d). This Pt NP enrichment in the tumor is consistent with our observations in patient tumors.

The secreted protein acidic and rich in cysteine (SPARC), which binds albumin, has an important role in the uptake of albumin-paclitaxel complexes^8^. We treated the DNR-resistant, K562-xenografted nude mice with Pt NPs and assessed, by immunofluorescence analysis, the spatial relationship between Pt NPs and SPARC in vivo. The Pt NPs were observed to co-localize with SPARC in tumor cells, which was also confirmed in DNR-resistant tumors (Supplementary Fig. 11). The HSA adsorbed onto the surface of the Pt NPs affords the NPs a tumor-targeting ability. However, whether the Pt NPs can cross the tumor vascular barrier and enter the tumor itself remained to be investigated. Therefore, we used an anti-SPARC antibody to label tumor vessels (green) to examine the localization of SPARC relative to that of Pt NPs labeled with ICG (indocyanine green; near infrared fluorescence probe). We observed a significant penetration of Pt NPs into the tumor tissue (Supplementary Fig. 12), demonstrating that the Pt NPs are able to overcome the bottleneck of crossing the vessel wall to transport a drug into tumors.

We proceeded to perform pull-down assays to investigate the possible interaction between SPARC and SA-coated Pt NPs. To this end, we incubated human- or mouse-derived Pt NPs with recombinant SPARC proteins. The protein-NP complexes were purified by ultracentrifugation and assayed by dot blot analysis, which revealed protein-protein interactions between SPARC and Pt NPs (Fig. 4e), suggesting that SPARC plays a key role in tumor targeting of the Pt NPs. Collectively, the dual tumor-targeting feature of the Pt NPs, comprising both passive targeting via the EPR effect and active targeting via albumin/SPARC signaling, makes the Pt NP a promising candidate for NP-mediated drug delivery.

To further track the tumor targeting dynamics of Pt NPs in vivo, we performed high-resolution radiotracer experiments. Human-derived Pt NPs were labeled with ^125^I and purified to generate ^125^I-Pt NPs. We directly injected 500 µCi ^125^I-Pt NPs into DNR-resistant K562-xenografted nude mice. The real-time radioactivity of ^125^I in vivo was tracked by nanoScan single-photon emission computed tomography (SPECT/CT). The radioactive signal accumulated in the tumor regions, peaking at 24 h and remaining apparent at 48 h P.I. (Fig. 4f), indicating that the Pt NPs were efficiently taken up by the tumors.

To determine whether the NPs remain coated with SA throughout the body, we performed time-dependent bio-distribution experiments using ^64^Cu-labeled NPs or ^64^Cu-labeled SA. The radiolabeled Pt NPs or SA was administered intravenously to C57BL/6 mice, which were sacrificed 4 h or 24 h P.I. The main organs were assessed for radioactivity by gamma counting. The amount of ^64^Cu-Pt NPs at 4 h and 24 h P.I. (29.84 ± 1.16% ID g^−1^ and 20.26 ± 1.03% ID g^−1^, respectively) in the blood was higher than that of ^64^Cu-SA (24.29 ± 1.44% ID g^−1^ and 10.76 ± 0.45% ID g^−1^, respectively; Fig. 4g), indicating that Pt NPs have a higher circulation half-life than SA. At 4 h P.I., the amount of ^64^Cu-Pt NPs in the spleen was higher than that of ^64^Cu-SA, but the kidney levels of ^64^Cu-Pt NPs were slightly lower than that of ^64^Cu-SA. These differences in spleen and kidney radioactivity between ^64^Cu-Pt NPs and ^64^Cu-SA may be due to the differences in size, which would lead to a stimulation of phagocytosis of the relatively large Pt NPs by splenic macrophages. At 24 h P.I., the difference in kidney levels between the two treatment groups decreased, suggesting that Pt NPs larger than 6 nm in size were slowly cleared through the GBM. These bio-distribution results are consistent with our observations of the glomerular filtration membrane ultrastructure.

**Fig. 5:**
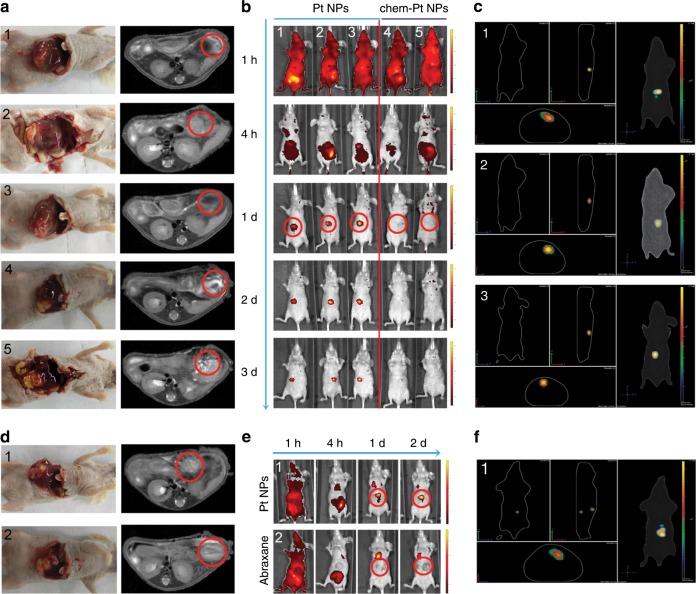
Orthotopic livertumor targeting of Pt NPs in vivo. **a** Anatomical location (left) and MIR imaging (right) of orthotopic liver tumors in 5 nude mice; **b** NIR image of tumors 1–3 d after intravenous injection with 1 mg kg^−1^ ICG-conjugated Pt NPs (1, 2, 3) or 1 mg kg^−1^ ICG-conjugated chem-Pt NPs (HSA prepared Pt NPs; 4, 5), **c** Three dimensional reconstruction of the NIR images of the orthotopic liver tumor models treated with 1 mg kg^−1^ ICG-conjugated Pt NPs (1, 2, 3); **d** Anatomical location (left) and MIR imaging (right) of the orthotopic liver tumors; **e** NIR image of a tumor 1–2d after intravenous injection with 1 mg kg^−1^ ICG-conjugated Pt NPs (1) or 1 mg kg^−1^ albumin bound Paclitaxol (Abraxane)-ICG (2) treatment. **f** Three dimensional reconstruction of the NIR image of the tumor after treatment with 1 mg kg^−1^ ICG-conjugated Pt NPs (1). All red circles indicate the tumor location.

Compared with commercially available albumin-prepared Pt NPs (chem-Pt NPs), we observed superior tumor targeting with the Pt NPs. The orthotopic liver tumor model (Fig. 5a) was treated with Pt NP-ICG or chem-Pt NP-ICG. The fluorescence in the chem-Pt NPs-ICG group was diffusely distributed throughout the body 1 h P.I.; the fluorescence disappeared almost completely at 24 h P.I. However, the fluorescence was still detected at the tumor site in the Pt NPs-ICG group at 24 h P.I. (Fig. 5b–c). The Pt NP fluorescence signal was retained for over 72 h. Under the same conditions, Pt NPs outperformed albumin-bound Paclitaxol in tumor targeting (Fig. 5d–f). One reason for this may be that without the protection of other proteins, single ingredient albumin is readily hydrolyzed, so the SA-coated NPs have a shorter in vivo half-life than Pt NPs (Supplementary Figs. 13 and 14). The protease inhibitor, α−2 macroglobulin^25^, in the protein corona of Pt NPs may prevent hydrolysis of the HSA prior to reaching the tumor site.

### Pt NPs reverse DNR resistance in tumor cells

Multidrug resistance, including a resistance to DNR, a primary chemotherapeutic, commonly occurs in patients. Therefore, there is an urgent need to develop a strategy to efficiently reverse drug resistance and improve the effectiveness of chemotherapy. One of the main mechanisms of drug resistance is an increase in intracellular GSH activity^9,26,27^, thus decreasing GSH levels is likely to reverse DNR resistance.

Since the Pt NPs target tumor cells in patients who undergo cisplatin treatment and Pt is able to spontaneously adsorb GSH, we hypothesized that Pt NPs can function as nanocarriers that improve the therapeutic efficacy of DNR by Pt-mediated reversal of resistance. We therefore investigated the anticancer potential of drug-loaded Pt NPs. We functionalized the Pt NPs with DNR, achieving drug loading by incubating the particles with drug in the presence of a high concentration of GSH (10 mM). GSH is able to reduce the disulfide bonds in albumin, opening its structure and exposing sulfhydryl groups to facilitate the DNR binding onto the Pt NP surface^7,26^. The spatial conformation of albumin was recovered by treatment with a low concentration of H_2_O_2_ to form a stable, drug-loaded system (DNR@Pt NPs; Supplementary Fig. 15a).The size of DNR@Pt NPs was 117nm, and zeta potentials of DNR@Pt NPs were −6.01 mV (Supplementary Figs. 16 and 17). The Pt NP DNR loading efficiency was approximately 31.5% (encapsulation efficiency was 24%), as determined by HPLC (Supplementary Fig. 15b-c). The DNR release rate was 72.8% in the presence of 5 mM GSH, by dialysis. Because the GSH concentration in human plasma and the extracellular environment is relatively low (2 μM), the spatial conformation of the albumin on Pt NPs remains stable until the NPs encounter the intracellular environment (2 mM GSH)^7,28,29^. At this concentration, the albumin can unfold to realize controlled drug release (Supplementary Fig. 15d).

The GSH concentration in DNR-resistant K562 cells was approximately 5.45 mM. As expected, intracellular GSH was consumed when the cells were exposed to Pt NPs (Supplementary Fig. 15e). The DNR (0.1 mg L^−1^) and DNR-Pt NPs (1 mg L^−1^) were taken up by drug-resistant K562 cells; the intracellular drug concentrations were 0.04 µg 10^−6^ cells and 0.12 µg 10^−6^ cells at 2 h P.I. As determined by CCK8 assay, the DNR@Pt NPs not only efficiently suppressed the growth of both DNR-sensitive K562 and DNR-resistant K562 cells, but also elicited a significant synergistic effect in tumor cytotoxicity, when compared to Pt NPs or DNR treatment alone (Supplementary Fig. 18). After exposure to DNR@Pt NPs, the drug resistance index of DNR-resistant K562 cells significantly decreased from 12.469 to 7.986 (Supplementary Table 3), indicating a partial reversal of drug resistance.

**Fig. 6:**
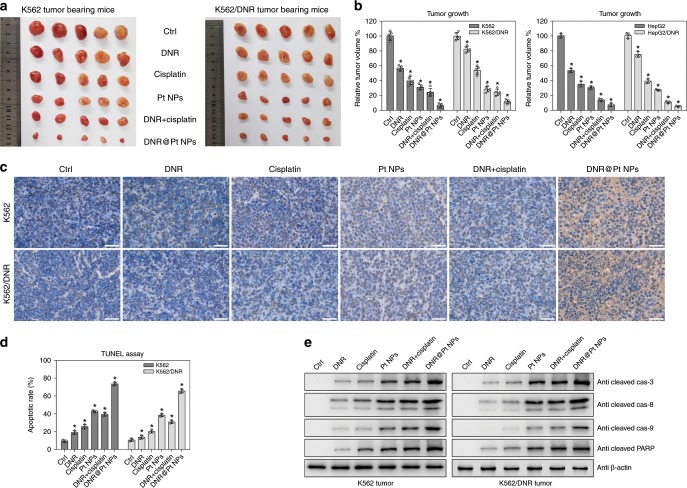
Pt NPs reverse the DNR resistance of tumor cells. **a** DNR-sensitive and DNR-resistant K562 cell tumor-bearing nude mice were treated with PBS (ctrl), 0.1 mg kg^−1^ DNR (DNR), 1 mg kg^−1^ cisplatin (cisplatin), 1 mg kg^−1^ Pt NPs (Pt NPs), 0.1 mg kg^−1^ DNR combined with 1 mg kg^−1^ cisplatin (DNR + cisplatin) or 1 mg kg^−1^ DNR-Pt NPs (DNR@Pt NPs). The tumors were excised and their volumes measured. **b** DNR-sensitive and DNR-resistant K562 cell (left, n = 5) or HepG2 cell (right, n = 3) tumor-bearing nude mice were treated as described in panel **a** and the tumor volumes were measured. * *P* < 0.05. **c** TUNEL staining of K562 tumor tissue sections. The staining labeled the nuclei of apoptotic cells brown and viable cells blue. Scale bar = 50 µm. **d** The percentage of apoptotic cells was determined (n = 3). * *P* < 0.05. **e** Western blot analysis of the levels of cleaved caspase-3, -8, and -9 in K562 tumor lysates. β-actin was detected as a loading control. Data = Mean + /− Standard deviation.

We next treated K562-xenografted nude mice with DNR, cisplatin, Pt NPs, DNR combined with cisplatin or DNR@Pt NPs separately (Fig. 6a). Compared with the untreated group, DNR treatment alone successfully inhibited tumor growth, suppressing tumor volume to ~56.61 ± 3.35% of the control. Treatment with Pt NPs alone also elicited a significant tumor volume reduction to ~31.18 ± 3.42% of the control. Treatment with DNR@Pt NPs resulted in a tumor volume of only 6.14 ± 2.52% of the control, which was better than the DNR and cisplatin combination group (24.24 ± 5.12%). Furthermore, the volumes of DNR-resistant K562 tumors treated with either DNR or cisplatin were decreased to 82.49 ± 3.72% or 53.72 ± 5.60%, respectively. The muted effect of the former is attributed to the drug-resistance. Importantly, the Pt NPs alone (28.34 ± 3.71%) showed a more effective anti-tumor activity than cisplatin. The tumors in mice treated with DNR@Pt NPs were significantly reduced in size (11.38 ± 2.67% of controls) than those treated with the individual formulations or combined drugs (Fig. 5b). When DNR (0.1 mg kg^−1^) or DNR@Pt NPs (1 mg kg^−1^) was administered to drug-resistant K562 tumor-bearing mice, the drug concentrations in the tumors were 0.6 and 2 µg g^−1^, respectively, at 24 h P.I. We also confirmed the enhanced anti-tumor effect of DNR@Pt NPs in human hepatocellular carcinoma (HepG2) xenograft-bearing (DNR-sensitive or -resistant) nude mice (Fig. 6b, Supplementary Fig. 19).

Terminal deoxynucleotidyl transferase dUTP nick end labeling (TUNEL) assay revealed that the apoptotic rate of K562 tumors was about 9.37 ± 1.38% in the control group (Fig. 6c). Treatment with DNR, cisplatin or Pt NPs increased the rate to 18.92 ± 1.88%, 25.66 ± 2.20% and 42.58 ± 1.37%, respectively, while treatment with DNR@Pt NPs dramatically increased the rate to 73.12 ± 2.08%, which was significantly higher than the DNR plus cisplatin group (39.09 ± 1.95%). Moreover, in DNR-resistant K562 tumors, apoptosis in the control group was 10.57 ± 1.66%. The fraction of apoptotic cells increased slightly with DNR (13.65 ± 1.98%) or cisplatin treatment (20.28 ± 1.67%), while the tumor apoptotic rate in the Pt NP and DNR@Pt NP groups increased to ~38.16 ± 1.63% and ~65.36 ± 2.21%, respectively (Fig. 6d).

Among all groups, the DNR@Pt NP group exhibited the greatest activation of cleaved caspase-3, -8 and -9, indicating activation of both the external death signal (caspase-8) and the mitochondria pathway (caspase-9)^30–32^. In DNR-resistant K562 tumors, the cleaved caspase-3, -8 and -9 signals in the DNR or cisplatin treatment group was relatively weak, while treatment with DNR@Pt NPs induced the highest level of cleaved caspase-3, -8, -9 (Fig. 6e). Our results demonstrate that Pt NPs can sensitize drug-resistant tumor cells to DNR treatment by decreasing intracellular GSH levels and inducing apoptosis.

### In vivo tolerability of the Pt NPs

Since Pt NPs larger than 6 nm are cleared at a lower rate than smaller Pt NPs, we investigated the consequences of the lengthy presence of these NPs in the body. To determine whether the Pt NPs may be hepatotoxic, we performed cytotoxicity assays using the human normal liver cell line L02. Compared to untreated cells, Pt NPs elicited little inhibition of cell growth, even at the highest dose of 10 µg mL^−1^ (Supplementary Fig. 20). The IC_50_ values of the Pt NPs in DNR-sensitive K562 cells, DNR-resistant K562 cells and the L02 cells (Supplementary Fig. 20, Table 3) were 3.67, 8.66 and 98.51 μg mL^−1^, respectively.

We further assessed the potential hepatotoxicity of Pt NPs in a zebrafish embryo model at 5 days postfertilization (dpf)^33,34^. Transgenic zebrafish embryos with GFP-expressing hepatocytes were monitored for systemic toxicity, with special attention to liver toxicity after the Pt NP treatment. We observed neither significant changes in the liver volume nor any morphological embryonic changes, compared to the untreated group (Supplementary Fig. 21a). We also performed the TUNEL assay in the zebrafish liver and observed no significant change in the number of apoptotic nuclei or cellular morphology after exposure to Pt NPs for 3–5 days (Supplementary Fig. 21b).

To verify the formulation long-term toxicity, we treated mice with Pt NPs for extended time periods, weighing the animals to evaluate toxicity. We observed no significant weight loss after 12 weeks of treatment (Supplementary Fig. 22). Moreover, histological examination revealed no apparent pathological changes or inflammation in the heart, liver, spleen, lung or kidney, after treatment for 12, 24 h and 12 weeks (Supplementary Figs. 23 and 24). We treated mice with Pt NPs and assessed IgM and innate immunity-related cytokines in the first 72 h (Supplementary Figs. 25 and 26). In addition, we found no significant changes in the activation of the main subsets of T cells, T helper cells (CD3^+^/CD4^+^) and cytotoxic T cells (CD3^+^/CD8^+^), by flow cytometry, after long-term Pt NP treatment of C57BL/6 mice (Supplementary Fig. 27). We also examined the activity of natural killer cell (NK, CD49b^+^/GranzymeB^+^), another type of immune cell, for 12 weeks. Again, Pt NPs exhibited favorable biocompatibility. Overall, our findings confirm that Pt NPs formed spontaneously in vivo are relatively harmless to mammals.

## Discussion

We have verified the presence of Pt NPs in the blood of patients treated with cisplatin, indicating the in vivo generation of Pt NPs. Our results reveal an interaction between HSA and cisplatin in the biosynthetic mechanism of in vivo Pt NP synthesis, and spontaneous interaction of Pt NPs with plasma proteins, to form what is known as a protein corona. Further, our findings facilitate the discovery of a previously unreported drug delivery system for cancer treatment. We found that Pt NPs can accumulate in tumors and remain for an extended period in the human body by their interaction with HSA. We systematically demonstrated the potential and feasibility of the Pt NPs as a drug delivery agent, capable of reversing DNR resistance by decreasing intracellular GSH levels and sensitizing DNR-resistant tumor cells to chemotherapeutic drugs. Other proteins (e.g., macroglobulin, apolipoproteins) in the corona formed on Pt NPs are favorable for tumor treatment and worthy of future study. As a biocompatible delivery system, the Pt NPs not only can target tumors, but also can be formed in vitro with a customized corona protein composition optimized for cancer treatment.

Since the first description of metal NPs in the 1960s, many metal NPs with unique properties have been created and numerous, groundbreaking studies on the effects of metal NPs in nature and medicine have been conducted. Despite these advancements, there has been very limited application of metal NPs in the clinic. The unknown potential toxicity, cost and ethical hurdles of research in humans have hampered the progress in this area. Our discovery of biosynthesized metal NPs from a clinically approved pharmaceutical in the human body help to bypass these obstacles. These phenomena that occur in the human body may be applicable to other metal formulations, which could then be further modified for applications in tumor treatment. In the case of Pt NPs, we combined the biosynthesis of metal NPs with an anticancer drug to form a combined therapeutic which has the potential to develop into personalized cancer medicines for patients with drug-resistant cancer.

## Methods

### Reagents

Cisplatin and its derivatives were purchased from Qilu Pharmaceutical Factory (China), glutathione (GSH), Cell Counting Kit-8/WST-8, 1-ethyl-3-[3-(dimethylamino) propyl] carbodiimide hydrochloride (EDC) and N-hydroxysuccinimide (NHS), Indocyanine Green (ICG), Daunorubicin (DNR) and chromatography reagents were purchased from Sigma (St. Louis, MO, USA). Primary antibodies, including those against human albumin (MAB1455, 1:500), human SPARC (AF941, 1:500) and mouse SPARC (MAB942, 1:500) were purchased from R&D Systems (MN, USA). Anti-mouse albumin (ab207327, 1:500) was purchased from Abcam (Cambridge, UK). Other antibodies, cleaved caspase-3 (#9664, 1:1000), -8 (#9496, 1:1000), -9 (#7237, 1:1000), PARP (#5625, 1:1000) and β-Actin (#4970, 1:3000) were purchased from Cell Signaling Technologies (Beverley, USA). For immune system assays, primary antibodies, including those against CD3-APC (565643, 1:200), CD4-FITC (557307, 1:200), CD8-PE (553033, 1:200), CD49b-FITC (553857, 1:200) and Granzyme B-PE (561142, 1:200) were purchased from BD (San Jose, USA). Mouse Th1/Th2/Th17 cytokine kit (560485) was purchased from BD (San Jose, USA). The horseradish peroxidase-labeled secondary antibodies were purchased from Molecular Probes (Eugene, USA). The terminal deoxynucleotidyl transferase-mediated dUTP nick end-labeling (TUNEL) kit was purchased from Roche Diagnostics (Mannheim, Germany). All aqueous solutions were prepared using Milli-Q ddH_2_O (Merck Millipore, Darmstadt, Germany). Other chemicals and solvents were used as received.

### Instruments

The following devices were used: TEM (JEM-1200EX, JEOL, Japan), EDS (QUANTAX, Bruker, Germany), UV-visible spectrophotometer (UV3600, Shimadzu, Japan), NTA (NanoSight NS300, Malvern, U.K.). The lattice fringe images were obtained by HRTEM (JEM-2100, JEOL, Japan) and further analyzed with Gatan digital micrograph software. Zeta potential was measured on a dynamic light scattering instrument (Zetasizer Nano ZS, Malvern, UK). Inductively coupled plasma mass spectrometry (ICP-MS, NexION, USA) was used to quantify the Pt contents. The imaging of dot blots or western blots was conducted on an enhanced chemiluminescence spectrometer (ChemDoc XRS, Bio-Rad, USA). Nanoflow electrospray ionization tandem mass spectrometric analysis of protein samples was carried out using an LTQ-Orbitrap Velos mass spectrometer (Thermo Scientific, Bremen, Germany) interfaced with an Easy-nLC II nano flow liquid chromatography system (Thermo Scientific, Odense, Southern Denmark).

### Cell lines and cell culture

The human chronic myelogenous leukemia K562 cell line and human hepatocellular carcinoma HepG2 cell line were obtained from the American Type Culture Collection (ATCC, Manassas, VA, USA). The DNR resistance index (RI) was calculated as the ratio of IC_50_ in the DNR resistant cells and DNR sensitive cells. The normal human liver cell line L02 was obtained from the cell bank of the Institute of Biochemistry and Cell Biology (Shanghai, China). The cells were cultured in Dulbecco’s Modified Eagle’s Medium (DMEM; Gibco, Grand Island, NY, USA) supplemented with 10% (v/v) fetal bovine serum (Gibco, Grand Island, NY, USA), 100 U mL^−1^ penicillin and 100 µg mL^−1^ streptomycin, at 37 °C in a humidified atmosphere containing 5% CO_2_.

### Tumor xenograft model and drug treatment

All animal experiments were conducted following the guidelines of the Animal Research Ethics Board of Nanjing Medical University (IACUC-10372). The tumor-bearing animal models were established by subcutaneous injection of 5 × 10^6^ tumor cells into ~4–6-week-old BALB/c nude mice weighing 20 ± 2 g (50% female and 50% male). The animals were obtained from Charles River Laboratories (Beijing, China), and kept in water and food ad libitum and a sustained environment at 24 ± 2 °C with a equal light/dark cycle. The volume of tumor was calculated with the equation *V* = (4/3)π[(*a* + *b*)/2]^3^, where *a* and *b* represents the smallest and largest diameter of the tumor respectively. When the was grown about 50 mm^3^, the mice were treated with the various formulations by intravenous injection 10 times within 20 days. We sacrificed animals and collected the tumor tissues for target analysis.

The HepG2 cells (10^7^/per mouse) were administered to the BALB/c nude mice. The mice were killed by an overdose of sodium pentobarbital, and the tumors were removed on day 7 after the operation. The tumors were cut into pieces (1 × 1 cm^3^). Anesthetize the nude mice and prepare for laparotomy. Localize the liver and make an incision in the liver. The tumors pieces (1 × 1 cm^3^) were put into liver incision. Hemostasis with cotton stopper and close the abdominal incision. The orthotopic HepG2 tumor mice can be detected by in vivo magnetic resonance imaging (MRI) imaging on day 7 after the operation.

### Human biological samples

Fresh blood was obtained from 100 patients (57 male/43 female) on cisplatin chemotherapy, fresh urine was obtained from 27 patients (15 male/12 female) on cisplatin chemotherapy and fresh feces was obtained from 10 patients (5 male/5 female) on cisplatin chemotherapy. All patients were from the Jiangsu Province Hospital; all human studies were performed with ethical review and approval by the Nanjing Medical University Management Committee and Ethics Committee (2017-647). The human tumor samples were obtained from patients on cisplatin chemotherapy who underwent surgical tumor resection.

### Separation and purification of Pt NPs

Two (2) mL blood samples from cisplatin chemotherapy patients were dispersed in 50 mL deionized water and centrifuged at 10^3^ g for 20 min to remove the insoluble cellular components, yielding an aqueous solution containing Pt NPs. The aqueous solution was centrifuged at different rates (varying from 10^4^*g* for 40 min to 10^5^*g* for 180 min), yielding samples of different sizes of Pt NPs. The samples containing Pt NPs were suspended in 0.5 mL deionized water for TEM analysis. The Pt NPs were further purified by HPLC (Hewlett-Packard Agilent 1100), with an ultimate XB-C18, 5 mm, 300 Å, 4.6 × 250 mm column (Welth, 00201-33043). The mobile phase was methanol-H_2_O-isopropyl alcohol (50%, 45% and 5%, pH8.0) with 0.1 mL min^−1^ flow rate. Each absorption peak was eluded and collected respectively, diluted and transferred for further characterization, including JEM-1010 TEM with oxford x-maxn EDS mapping and UV-VIS analysis. The amount of Pt NPs in each sample was quantified by ICP-MS. Moreover, the Pt NPs from patients’ urine (10 mL from each individual) and feces (10 g each person) were separated and purified as described above.

### Synthesis of Pt NPs from human serum and cisplatin in vitro

In general, 0.3 mg cisplatin was dissolved in 50 mL ddH_2_O to which the different reaction components were added, i.e., 2 mL human serum, cells centrifuged from 2 mL peripheral blood or 0.5 g human serum albumin. To verify the role of platinum in the synthetic process, EDTA (0.01 mol L^−1^) was added to the appropriate reactions. The resultant solution was stirred for 48 h at 37 °C, followed by differential centrifugation varying from 10^4^*g* for 40 min to 10^5^*g* for 180 min. The sedimented Pt NPs obtained by centrifugation were collected and resuspended for TEM analysis. The concentration of Pt NPs was quantified using ICP-MS.

On a standard chemotherapy regimen, the adult cisplatin dosage is 90 mg m^−2^ (1.7 m^2^ is typically used for adults), and the blood volume of an adult is 4–5 L. Therefore, the normal dose is 40 mg L^−1^ blood = 0.04 mg mL^−1^, so we used a cisplatin concentration of 0.04–0.1 mg mL^−1^ to investigate the biosynthesis of Pt NPs in vitro. In order to synthesize a certain amount of Pt NPs for the study of the anti-cancer effect, 2 mg cisplatin was dissolved in 20 mL ddH_2_O to which the 20 mL human serum were added, and the resultant solution was gently shake for 48 h at 37 °C, followed by differential centrifugation varying from 10^4^*g* for 40 min to 10^5^*g* for 180 min.

### In vivo biosynthesis of Pt NPs

C57BL/6 mice were purchased from Charles River Laboratories (Beijing, China) and maintained in a controlled environment at 24 ± 2 °C with a 12 h light/dark cycle and received food and water ad libitum. For the in vivo synthesis of Pt NPs, the animals were treated with cisplatin in three ways: (1) fed a 0.01 mg mL^−1^ aqueous solution of cisplatin for 48 h, (2) intravenously administered cisplatin (30 mg kg^−1^) or (3) intraperitoneally injected with cisplatin (50 mg kg^−1^). The animals were then sacrificed, and blood, liver, bile and feces sample were collected. The Pt NPs were separated by differential centrifugation and purified by HPLC as described above for human samples.

### Dot blot assay

The protein samples were gently pointed onto PVDF membrane until dried at 25 °C. The membrane was incubated with the primary antibody at 4 °C overnight, followed by incubation with a horseradish peroxidase-labeled secondary antibody at room temperature for 2 h. The protein levels were visualized by chemiluminescence (ECL, Pierce, USA). The original bolt images are supplied in Source Data file.

### SDS-PAGE electrophoresis

The Pt NPs, chem-Pt NPs, albumin bound Paclitaxel nanoparticles (20 µL) were loaded and run in a 10% separating gel and 5% stacking gel (Sigma, USA) for 1 h. The gels were stained with coomassie brilliant blue (Sigma, USA). The original gel images are supplied in Source Data file.

### MS assay of protein for Identification of protein corona

A solution of 5 mg mL^−1^ was prepared by adding ultrapure water to each sample. We used the filter-aided sample preparation (FASP) method^35^ to prepare digested samples for protein identification with 200 µL of each sample. After reduction and alkylation, the solution was buffer-exchanged with 50 mM NH_4_HCO_3_ (pH 8.0–8.5) using 10 kDa molecular weight cut-off Amicon Spin Tube (Millipore, MA). For digestion, 4 µg sequencing-grade modified trypsin (Promega, USA) was added to each solution, followed by a 37 °C overnight incubation. Prior to LTQ-Orbitrap analysis, the peptide preparations were desalted using Ziptip C18 cartridges (Millipore, MA) and reconstituted with 0.1% formic acid (FA).

For lipidomic analysis, the 250 µL pre-cooled chloroform/methanol was added to 50 µL of each sample. The mixtures were vortexed for 1 min and allowed to react for 1 h. After a 10 min centrifugation at 14,000*g*, the supernatant was evaporated to dryness and the preparation was reconstituted with chloroform/methanol (1:1, v/v) prior to qTOF analysis.

The samples were assessed using a DIONEX nano LC system (Thermo Scientific), coupled to an LTQ-Orbitrap mass spectrometer (Thermo Scientific), to identify proteins. We carried out the chromatographic separation using a C_18_ column (Acclaim PepMap RSLC, 75 μm × 50 cm, 3 μm, 100 Å, Thermo Scientific) using a linear gradient of 3-35% buffer (acetonitrile with 0.1% FA) at a flow rate of 0.3 μL min^−1^ over 132 min. We employed a 2.2 kV ionization voltage for positive detection and selected the five most prominent ions from the 60,000 resolution MS full scan (m/z 350–1800) for MS/MS analysis, provided that they were at least doubly charged. The normalized collision energy for HCD (higher energy collision induced dissociation) was set to 35%. Before analysis, a standard solution was used for mass calibration. The raw data acquired by the Nano LC-LTQ-Orbitrap were imported into Maxquant software (Max-Planck-Institute of Biochemistry, Germany)^36^ to identify and quantify the proteins. The following parameters were used: trypsin for enzyme digestion; oxidation of methionine as variable modification; a precursor mass tolerance of 20 ppm; a fragment mass tolerance of 0.02 Da. The human reference database (173260 protein sequences) was downloaded from UniprotKB and used as the searching database. The identification criterion for peptides was a <1% FDR value according to the database searching. iBAQ quantification was enabled^37^.

Next, we quantified the contents of several main proteins by ELISA. The mass ratio of HSA, macroglobulin, apolipoprotein was checked by Albumin Human ELISA Kit (Abcam, UK), alpha2 Macroglobulin Human ELISA Kit (Abcam, UK) and Human Apolipoprotein A1 ELISA Kit (Thermo, USA), respectively.

### Pt NPs crosslink ICG

Briefly, 5 mg of ICG (current FDA approved NIR fluorophores) was dissolved in DMSO (2 mL) followed by 3 mg of EDC and 2 mg of NHS was mixed in 10 mL ddH_2_O by inversion at room temperature in the dark overnight. Then 20 mg of Pt NPs were slowly added and mixed by inversion at room temperature for 10 h in the dark. The Pt NPs-ICG were dialyzed against distilled water using a dialysis tube and lyophilized.

### Immunofluorescence assay

K562 cells, and DNR-sensitive and -resistant tumor-bearing mice were treated with Pt NPs. The tumor sections were incubated with an Alexa 594-conjugated anti-human albumin antibody and an Alexa 488-conjugated anti-SPARC antibody. The localization of SPARC and Pt NPs was imaged and analyzed by confocal laser scanning microscopy (LSM710, Carl Zeiss, Germany). The DNR-resistant tumor-bearing mice were treated with ICG-conjugated Pt NPs. Tumor sections from the mice were incubated with an Alexa 488-conjugated anti-SPARC antibody. The localization of SPARC and ICG-conjugated Pt NPs was imaged and analyzed by confocal laser scanning microscopy (LSM710, Carl Zeiss, Germany).

### Application of Pt NPs for in vivo imaging

Orthotopic HepG2 tumor-bearing mice were treated with ICG-conjugated Pt NPs, ICG-conjugated chem-Pt NPs (HSA prepared Pt NPs) or ICG-conjugated Albumin bound Paclitaxol (Abraxane) by intravenous injection. At different time points, the animals were anesthetized. Tumor NIR images were acquired and analyzed by a Xenogen IVIS Spectrum system (Caliper, USA).

### Pull-down assay

A solution of human blood-derived Pt NPs (1 mg mL^−1^) was incubated with recombinant human SPARC (R&D Systems, MN, USA) proteins at a concentration of 100 µg mL^−1^, 50 µg mL^−1^ or 10 µg mL^−1^. After incubation at 4 °C for 4 h, the SPARC-Pt NP complexes were sedimented by ultra-centrifugation. The level of human SPARC protein was analyzed by dot blot analysis; human albumin in the Pt NPs was also analyzed as a loading control. To examine the interaction between mouse-derived Pt NPs and mouse SPARC, the same protocol was applied as described above by incubating with recombinant mouse SPARC (R&D Systems, MN, USA) protein. The original bolt images are supplied in Source Data file.

### In vivo nanoScan SPECT/CT imaging

Pt NPs (100 µg) and Na^125^I (2.0 mCi; Beijing Atom High Tech, Beijing, China) were mixed in phosphate buffer (0.1 M, pH 7.4) in an Iodogen-coated vial (40 µgI odogen; Sigma, St. Louis, MO). After 10 min incubation at room temperature, a PD MiniTrap G-25 column (GE Healthcare, Buckingmashine, UK) was used to purify the iodinated NPs. The MiniTrap G-25 column was washed with 6 mL phosphate buffer and saturated with 4 mL 1% BSA before purification. The MiniTrap G-25 column was loaded with ~100 µL NP suspension and then washed with 3 mL phosphate buffer. The 0.5 mL fraction between 0.75 and 1.25 mL of the eluent was retained. To evaluate the radiochemical purity (RCP) of the labeled NPs, we performed instant thin-layer chromatography (ITLC) on silica-gel paper strips (GelmanSciences, Ann Arbor, MI), using acetone as the developing solution. We resuspended the radiolabeled Pt NPs in saline to 500 µCi 0.1 mL^−1^ for imaging experiments in animals.

For the in vivo, whole-body nuclear imaging experiments, tumor-bearing mice (18–20 g) were administered a tail vein injection of 500 µCi ^125^I-Pt NPs. Images were then acquired at 1, 4, 24 and 48 h P.I. The mice were anesthetized with 1.5% isoflurane and 0.5–1 L per minute oxygen during the imaging, which was performed on a nanoScan SPECT/CT in vivo preclinical imager (Mediso, Budapest, Hungary), fitted with 2 mm pinhole collimators, in helical scanning mode (20 projections, 30 min). The CT projections were recorded in the cone-beam CT subsystem of the instrument at 55 kV tube voltage and 170 µA current, with an isotropic voxel size of 72 µm. All images were reconstructed with Nucline software (Mediso Imaging System, Budapest, Hungary).

### Biodistribution of Pt NPs

NOTA-conjugated Pt NPs were initially prepared as follows: A suspension of 2 mg Pt NPs was combined with 0.3 μmol maleimido-mono-amide-NOTA (Macrocyclics, Dallas, TX) in PBS (0.1 M, pH 7.4) and incubated for 3 h at room temperature with stirring. The reaction mixture was then ultrafiltered at 4 °C and purified with a PD MiniTrap G-25 column to remove any excess maleimido-mono-amide-NOTA. The same method was used to generate the NOTA-SA control. Next, 200 µg NOTA-Pt NPs was suspended in 100 µL ammonium acetate buffer (0.5 M, pH 5.1), after which ^64^CuCl_2_ (5 mCi; 10 µL in 0.05 MHCl) was added and the mixture incubated at 40 °C for 30 min with shaking. Purification was performed with a PD MiniTrap G-25 column which was used to purify the NPs; the purity was assessed by ITLC as described above. For animal experiments the formulations were prepared by resuspending the purified ^64^Cu-Pt NPs in saline to a concentration of 40 µCi 0.1 mL^−1^.

The C57BL/6 mice (~4–6 weeks old) were randomized into four groups of four animals. Each mouse received ^64^Cu-SA or ^64^Cu-Pt NPs (40 µCi in 0.1 mL saline; i.v.). The animals were anesthetized with an intraperitoneal injection of sodium pentobarbital (45.0 mg kg^−1^). The mice were sacrificed at 4 and 24 h P.I. and the blood, heart, liver, spleen, kidney, stomach, intestine, bone, muscle and brain were harvested and weighed and the radioactivity was measured in a WIZARD automatic gamma counter (Perkin Elmer, Waltham, MA).The results are presented as the percentage injected dose per gram of tissue (%ID g^−1^).

### GSH consumption assay

Cells (2 × 10^6^) were seeded in 10-cm Petri dishes and grown overnight. The samples were then treated with a series of concentrations of Pt NPs for 12 h. Reductive glutathione (GSH) was detected using the Sigma-Aldrich Glutathione Assay Kit (CS0260). The GSH consumption (%) was calculated as the ratio of GSH in the Pt NP-treated cells to the PBS-treated control cells.

### Preparation of DNR-loaded Pt NPs

DNR (1 mg, 1 mg mL^−1^) was generally dropped to a Pt NP solution (1 mg, 1 mg mL^−1^) under the condition of 10 mM GSH under mixing in 40 mL. The reaction solution was reacted 48 h to for the synthesis of DNR-loaded Pt NPs. The solution was centrifuged at 10^5^*g* for 180 min. The precipitate, containing the DNR-loaded Pt NPs, was collected and washed with 0.1% H_2_O_2_ for 10 min to remove the reductive GSH. The DNR-loaded Pt NPs were then freeze-dried until further use.

### DNR loading rate and in vitro release profile

DNR-loaded Pt NPs were added in water (1 mL) and extracted in chloroform (1 mL). Next, HPLC method (Agilent 1100 equipped with a Kromasil 100-5C18 analytical column) was established and 10 μL of the solution was determined. The mobile phase, 5% triethylamine in 90% methanol (v/v), and the 230 nm excitation wavelength was selected. Then, we calculated the DNR loading rate (DL): DL% = Ws/Wp, the Ws and Wp indicated the weight of DNR and DNR-loaded NPs, respectively.

The drug release assays were carried out at pH 6.0 (pH of the tumor micro environment). 10 mg DNR-loaded Pt NPs was suspended in 5 mL PBS and transferred to a dialysis bag. The dialysis bag was then immersed in 95 mL PBS, pH 6.0. The release medium was vortex at 37 °C with a magnetic stir at 50 RPM continuously. For checking GSH concentration, 2 mL of the external medium was collected and replaced with the 2 mL of fresh PBS after 12 h. The amount of DNR released into the medium was determined by HPLC.

After DNR and DNR@Pt NPs were taken into drug resistant K562 cells and drug resistant K562 tumor mice, the intracellular drug concentrations were determined by HPLC.

### Potential antitumor activity and TUNEL staining

K562-xenografted nude mice or DNR-resistant K562-xenografted nude mice were administered DNR, cisplatin, Pt NPs, DNR combined with cisplatin or DNR@Pt NPs to assess the antitumor activity of the NPs. Apoptosis in tumor tissue sections was measured by terminal deoxynucleotidyl transferase mediated dUTP nick end-labeling (TUNEL) or by Klenow DNA fragmentation detection (Roche, Indianapolis, IN, USA) after permeabilizing the sections with 20 μg mL^−1^ protease K and removing any endogenous peroxidase with 3% H_2_O_2_ (in methanol). In the latter method, the 3′-OH ends of the fragmented DNA are labeled with biotin-dNTP via the Klenow fragment at 37 °C for 1.5 h. The tumor section slides were then incubated with streptavidin-HRP, followed by incubation with DAB. Apoptotic cells were identified as having dark brown nuclei by light microscopy (Axio Imager, Carl Zeiss, Germany). Ten images were acquired from three randomly selected sections from each tumor and evaluated for statistical analysis.

### Toxicityof Pt NPs in developing Zebrafish embryos

Zebrafish (*Danio rerio*) of the *lfabp:GFP* transgenic line were raised and maintained in 30 L tanks at 28.8 °C with a 14 h:10 h light/dark cycle. The successful breeding was followed, and eggs were subsequently collected from the bottoms of the tanks through a mesh. The Pt NPs (1 mg L^−1^) were added to the Zebrafish embryos for 3–5 days to assess toxicity.

### Toxicity of Pt NPs in mice

To examine long-term toxicity, we treated mice (C57BL/6 mice) with or without Pt NPs for 12 weeks and monitored body weight and any pathological changes or inflammation in the heart, liver, spleen, lung and kidney. Histological analysis of C57BL/6 mouse heart, liver, spleen, lung, and kidney after 1 mg kg^−1^ Pt NP treatments for 12, 24 h and un-treated were performed. The immune-related cytokines in the blood were also assessed. To evaluate the biocompatibility of Pt NPs, in terms of immunogenicity, the mice (C57BL/6) were treated with or without Pt NPs, after which the levels of IgM and innate immunity-related cytokines were assessed during the first 72 h.

### Statistical analysis

All results are presented as the mean ± standard deviation. The Student’s t-test was performed to compare groups at each time point. A value of *P* < 0.05 was considered statistically significant.

### Reporting summary

Further information on research design is available in the Nature Research Reporting Summary linked to this article.

## Supplementary information

Supplementary Information

Reporting Summary

## Data Availability

The authors declare that all data supporting the findings of this study are available within the paper, Supplementary Information and Source Data files. For further request please contact the corresponding author.
