# Supplementary material for: Blood-triggered generation of platinum nanoparticle functions as an anti-cancer agent
Source: Nat Commun. 2020 Jan 28;11:567. doi: 10.1038/s41467-019-14131-z (PMC6987201; doi:10.1038/s41467-019-14131-z)
Supplement: Supplementary file 1 — Supplementary Information [file 41467_2019_14131_MOESM1_ESM.pdf]

Supplementary Information

Blood-triggered generation of platinum nanoparticle functions as an anti-cancer agent

Zeng et al.

Supplementary information

Blood-triggered generation of platinum nanoparticle functions as an anti-cancer agent

Xin Zeng[†], Jie Sun[†], Suping Li[†], Jiyun Shi[†], Han Gao, Wei Sun Leong, Yiqi Wu, Minghui Li, Chengxin Liu, Ping Li, Jing Kong, Yi-Zhou Wu[*], Guangjun Nie[*], Yuming Fu[*] and Gen Zhang[*]

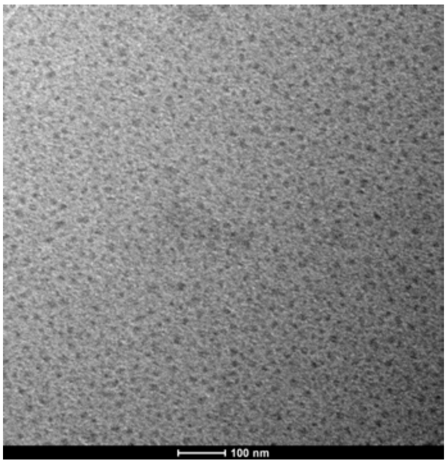

Supplementary Figure 1. Biological electron microscopy (120 kV) image of Pt NPs.

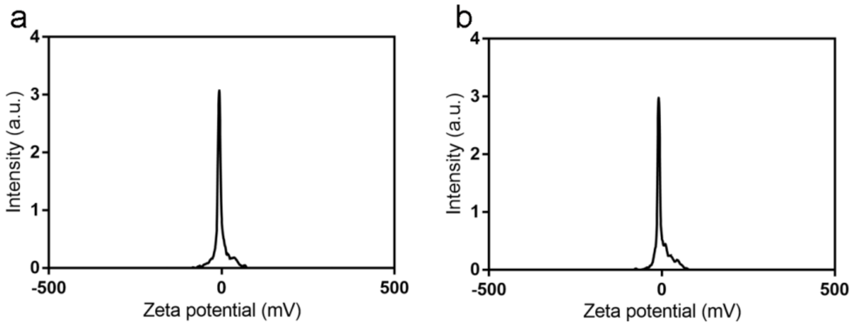

Supplementary Figure 2. Zeta potentials of Pt NPs in PBS buffer (a) and FBS (b).

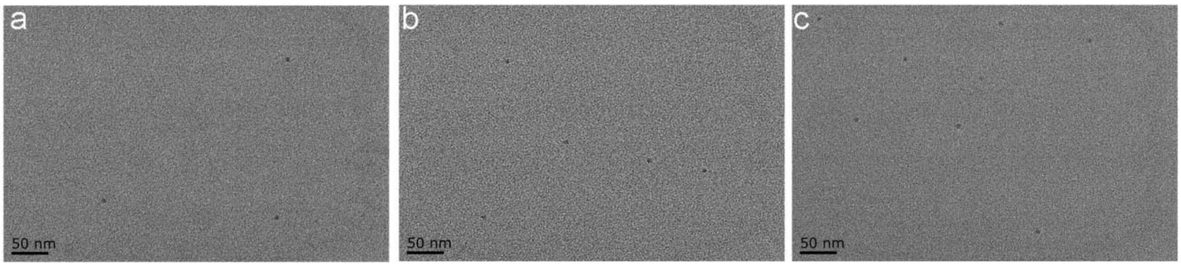

Supplementary Figure 3. Pt NP formation from platinum-based chemotherapy agents in patient blood. **(a)** Representative TEM image of Pt NPs from carboplatin in patient blood for 24 h. **(b)** Representative TEM image of Pt NPs from oxaliplatin in patient blood for 24 h. **(c)** Representative TEM image of Pt NPs from nedaplatin in patient blood for 24 h.

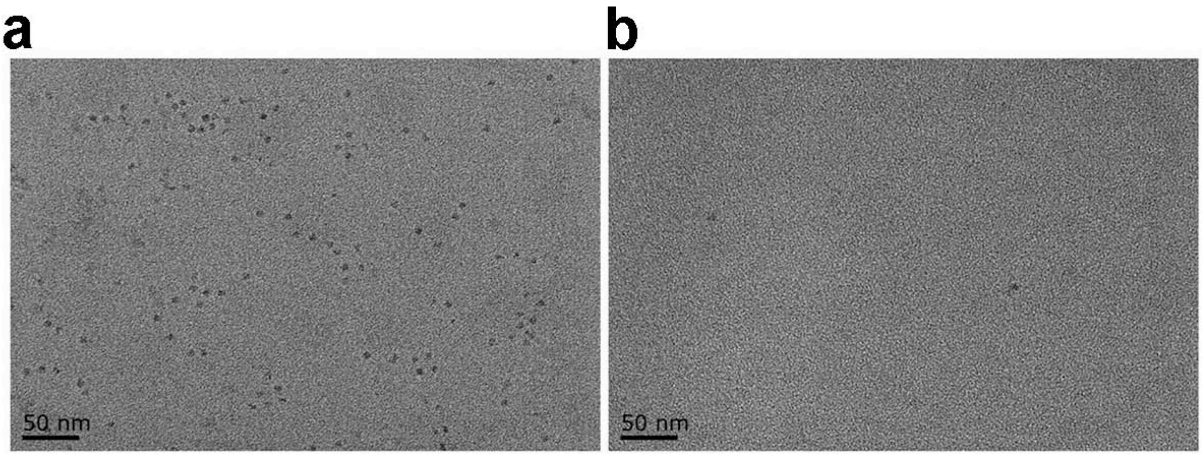

Supplementary Figure 4. Effect of metal chelation on Pt NP formation from cisplatin and serum albumin (SA). **(a)** Representative TEM image of a reaction sample after SA (0.5 g) and cisplatin (0.3 mg) were incubated in 50 mL ddH_2O at 37°C without the addition of a chelator. **(b)** Representative image of a sample prepared as in panel a, supplemented with EDTA (0.01 mol L^{-1}).

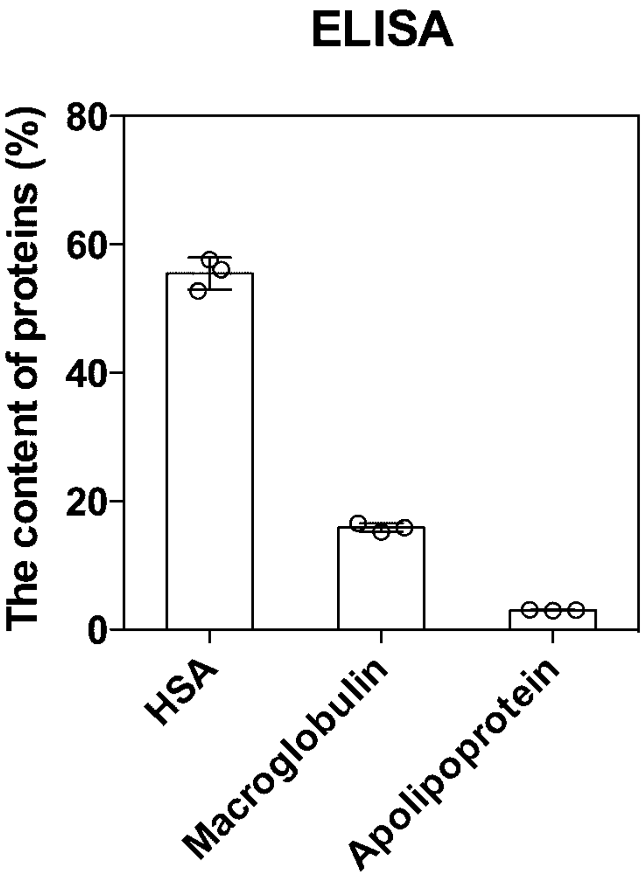

Supplementary Figure 5. Quantification of the major Pt NP-associated proteins by ELISA. Data = Mean +/- Standard deviation (n=3).

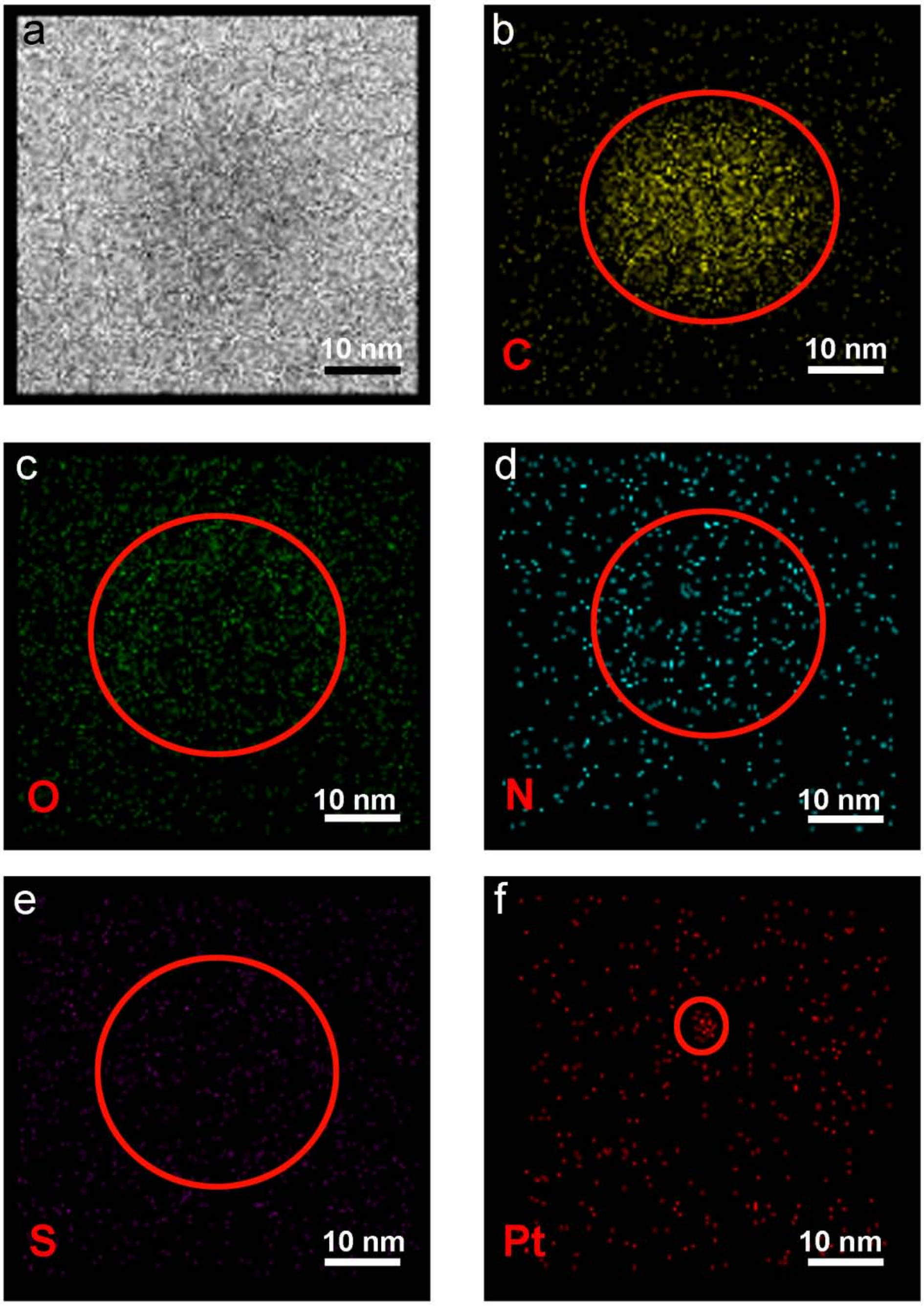

Supplementary Figure 6. Chemical composition of Pt NP. **(a)** A bio-TEM image of Pt NP. **(b-f)** EDS mapping of a single Pt NP.

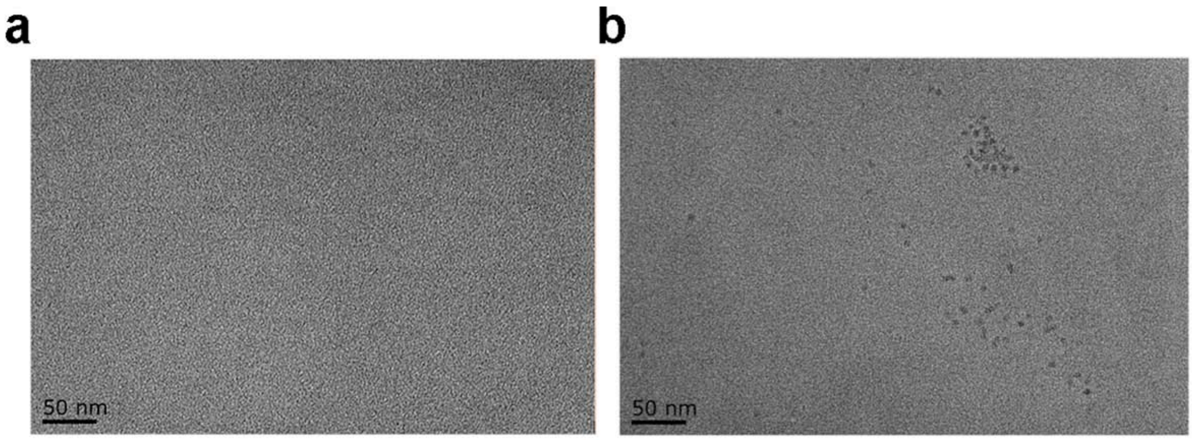

Supplementary Figure 7. Pt NPs form in patient serum. **(a)** Representative TEM image of lysates from patient peripheral blood cells isolated from 2 mL blood, cultured in serum-free medium and incubated with cisplatin (0.3 mg) for 48 h. **(b)** Representative TEM image of patient serum used in place of blood cells as in panel a.

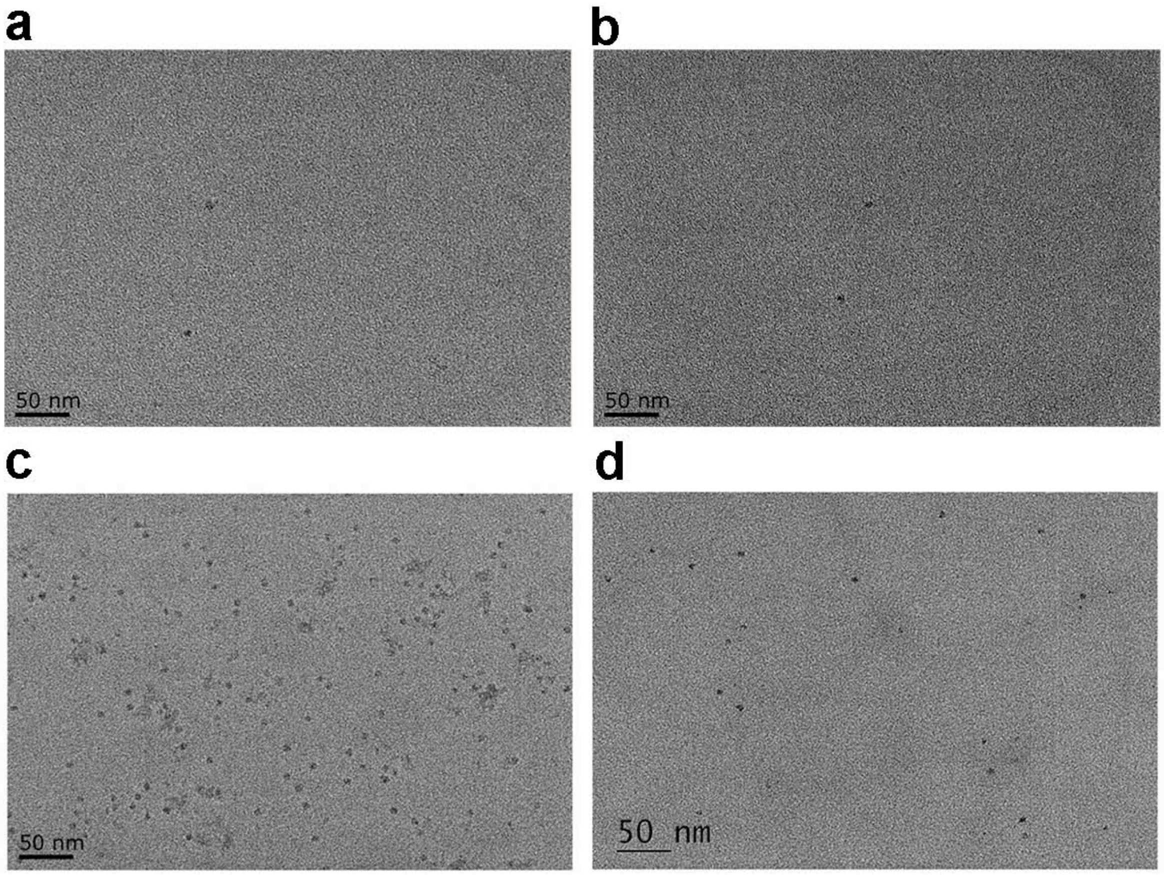

Supplementary Figure 8. Excretory pathways of *in vivo*-formed Pt NPs. **(a-b)** 24 h after cisplatin chemotherapy the urine **(a)** and the feces **(b)** from patients were collected and extracted to reveal low concentrations of Pt NPs (>6 nm). **(c-d)** After intravenous cisplatin (2 mg kg^{-1}) treatment of mice, Pt NPs (>6 nm) were present in **(c)** the liver and **(d)** the gallbladder.

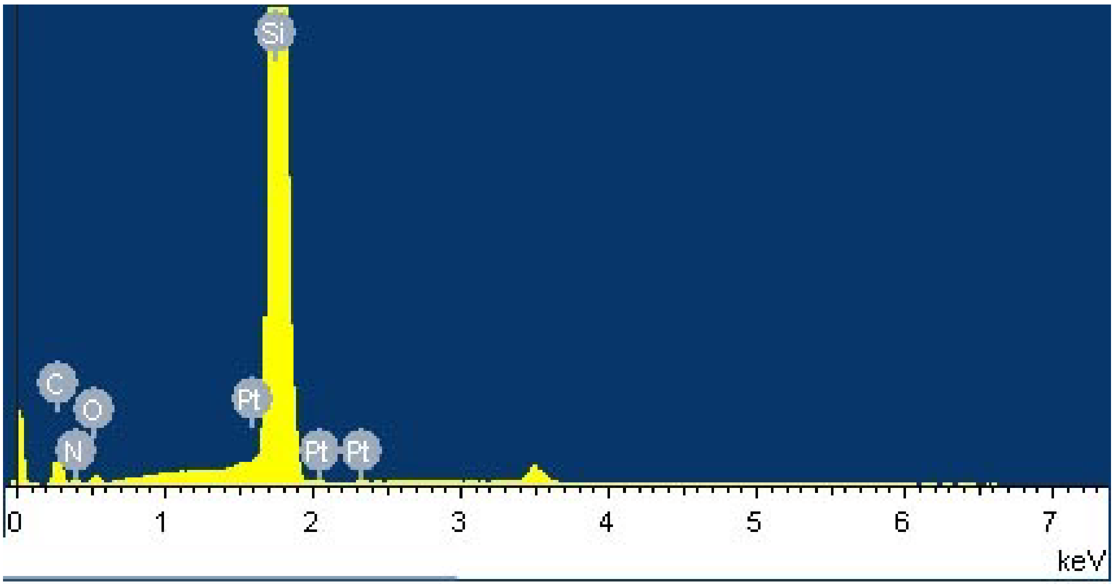

Supplementary Figure 9. EDS analysis of Pt NPs isolated from human tumor tissue.

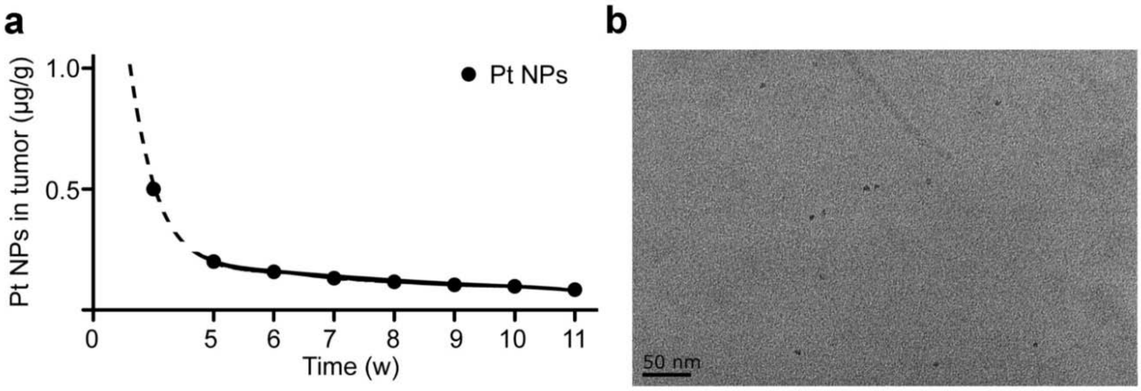

Supplementary Figure 10. (a) ICP-MS analysis of the concentration of Pt NPs in human tumors and **(b)** TEM analysis of Pt NPs in K562 tumor tissue.

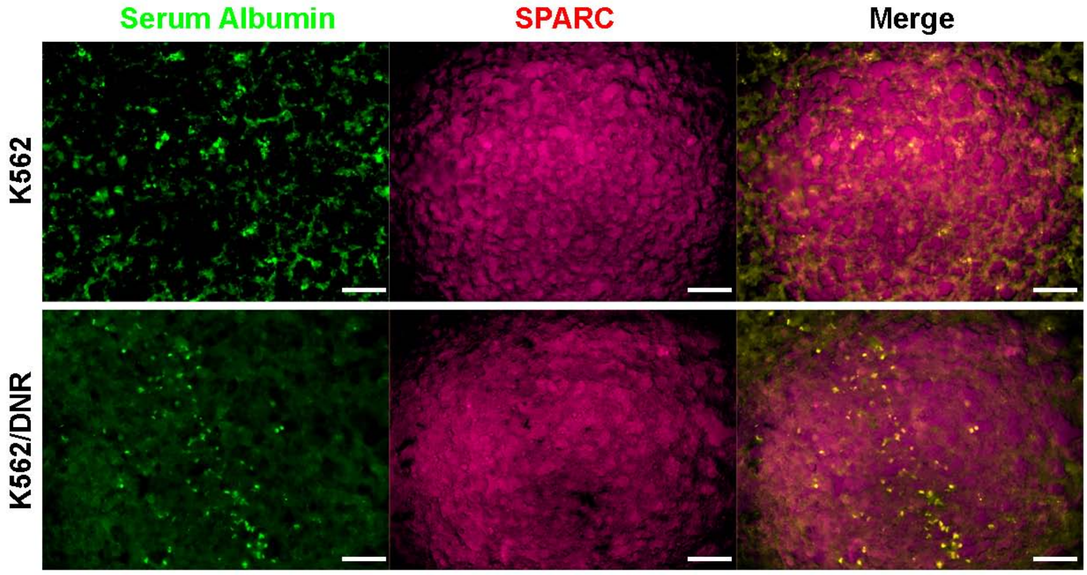

Supplementary Figure 11. Representative immunofluorescence images of tumor sections from DNR-sensitive (upper panels) and-resistant (lower panels) K562 cell-xenografted mice treated with 2 mg kg^{-1} Pt NPs. The tumor sections were stained with Alexa 594-conjugated anti-human albumin antibodies (green) and Alexa 488-conjugated anti-SPARC antibodies (red). Scale bar = 50 μm.

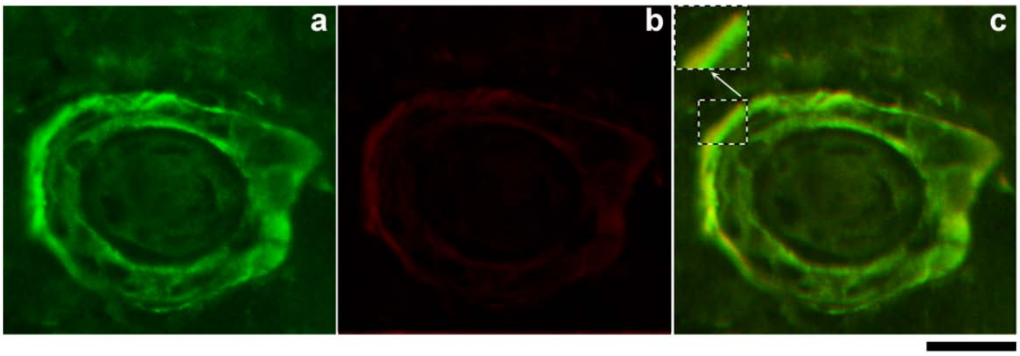

Supplementary Figure 12. Representative immunofluorescence images of tumor sections from resistant K562 cell-xenografted mice treated with 2 mg kg^{-1} Pt NP-ICG. The tumor vasculature was stained with Alexa 594-conjugated anti-SPARC (a, green) and Pt NP-ICG (b, red), the merged image is presented in c. Scale bar = 5 μm.

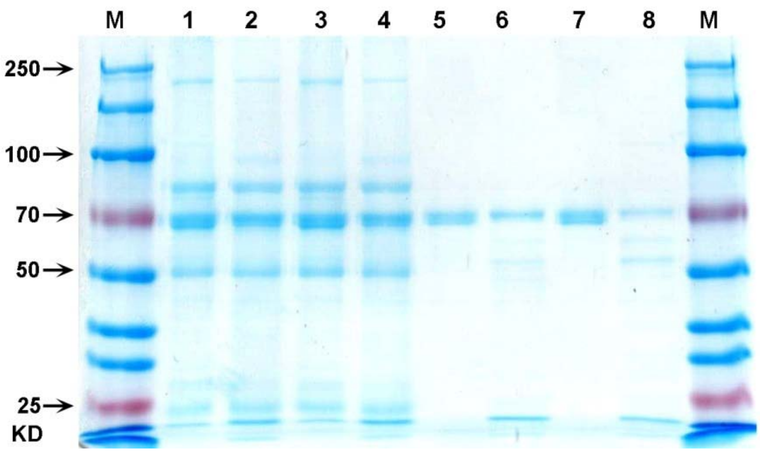

Supplementary Figure 13. Analysis of proteins in Pt NPs and chem-albumin Pt NPs after trypsin digestions for different time intervals. Coomassie Brilliant Blue stained SDS-PAGE gel of Pt NPs (1 μg) and chem-albumin Pt NPs (1 μg), 1. Pt NPs from whole blood, 2. Pt NPs after 2 min trypsin, 3. Pt NPs from whole blood, 4. Pt NPs after 10 min trypsin, 5. chem-albumin Pt NPs, 6. chem-albumin Pt NPs after 2 min trypsin, 7. chem-albumin Pt NPs, 8. chem-albumin Pt NPs after 10 min trypsin. M, Mw (molecular weight) standard

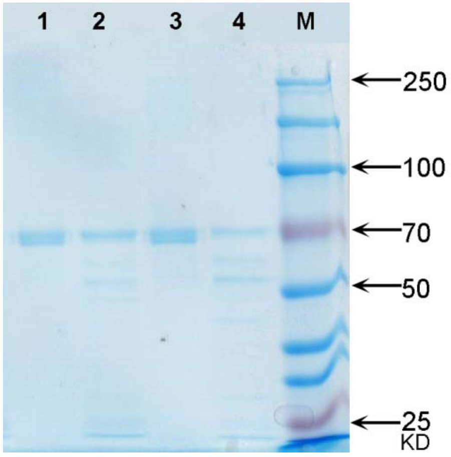

Supplementary Figure 14. Analysis of proteins in albumin-bound Paclitaxel nanoparticles after trypsin digestion for different durations. Coomassie Brilliant Blue stained SDS-PAGE gel of albumin-bound Paclitaxel NPs (1 μg). 1. Albumin-bound Paclitaxel NPs, 2. Albumin-bound Paclitaxel NPs after 2 min trypsin, 3. Albumin-bound Paclitaxel NPs, 4. Albumin-bound Paclitaxel NPs after 10 min trypsin. M, Mw (molecular weight) standard.

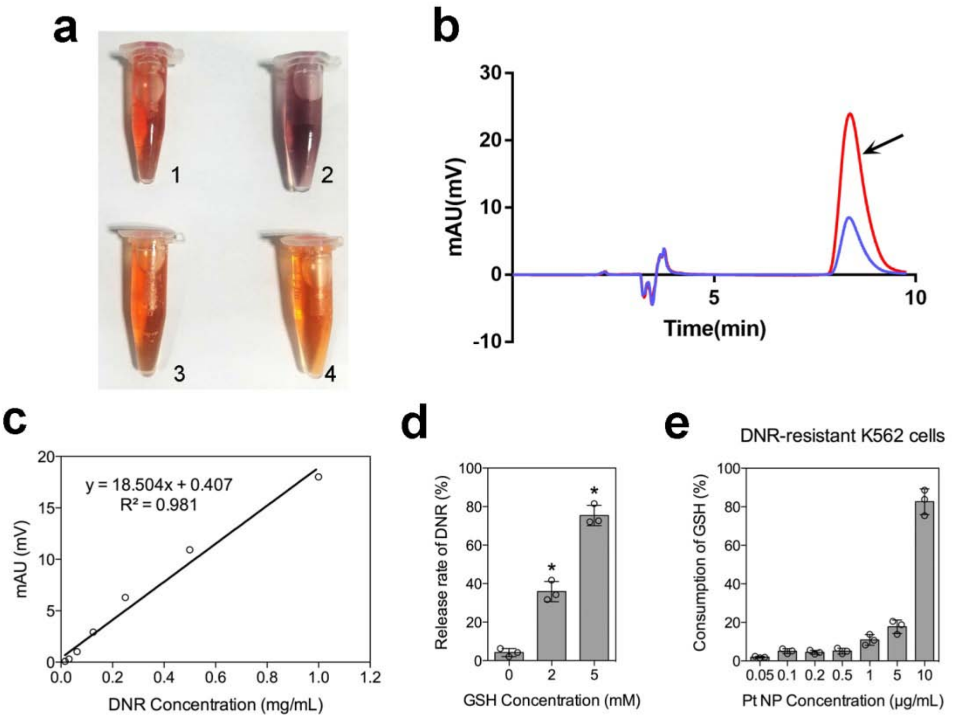

Supplementary Figure 15. Loading of Pt NPs with DNR. **(a)** 1 mg Pt NPs was added to an aqueous solution of DNR (1 mL, 1 mg mL^{-1}) and imaged after 0 h (1) and 12 h (2); two more samples were prepared similarly with the addition of 10 mM GSH and imaged at 0 h (3) and 12 h (4). **(b)** Drug loading (DL) rate was analyzed by HPLC. The retention time of a standard DNR solution at 0.1 mg mL^{-1} (red line) was approximately 8.868 min (arrow); DNR in the DNR-loaded Pt NPs, blue line. **(c)** Standard curve generated from different concentrations of DNR. According to the standard curve, the DNR loading efficiency (b) onto the Pt NPs was calculated to be 31.5%. The mass fractions of Pt NPs and DNR in DNR@Pt NPs were calculated to be approximately 76% and 24%, respectively. **(d)** The DNR release rate was analyzed by HPLC. To determine the drug release rate, a 0.1 mg mL^{-1} pure DNR solution was used as a standard. The bars in the graph represent the results from three independent experiments. Significant difference compared with the 0 group (*P<0.05). **(e)** GSH consumption assay in DNR-resistant K562 cells. The cells were treated with the indicated concentrations of Pt NPs and assayed after 12 h. Data = Mean +/- Standard deviation (n=3).

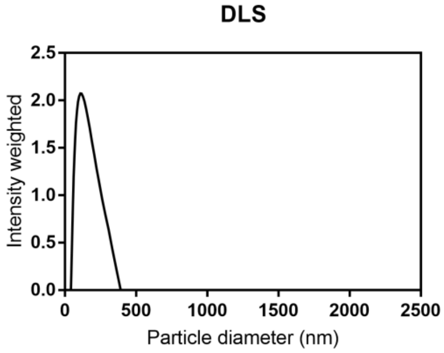

Supplementary Figure Fig. S16. The hydrated state size of DNR@Pt NPs in PBS (117 nm)

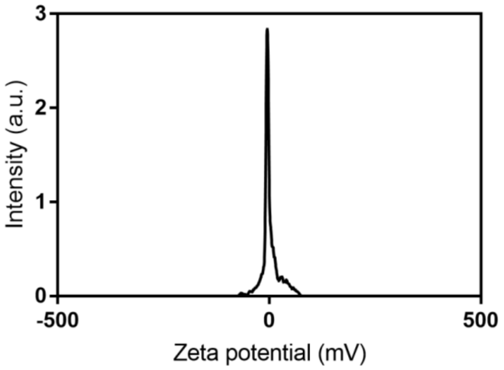

Supplementary Figure 17. The zeta potential of DNR@Pt NPs in PBS (-6.01 mV).

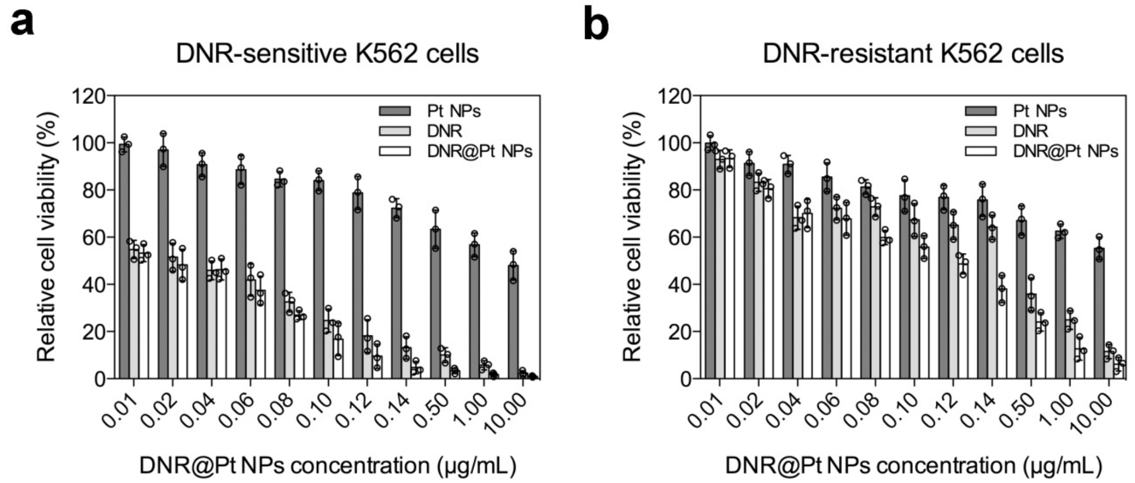

Supplementary Figure 18. Cell Counting Kit (CCK-8) assay for cell viability in K562 cells **(a)** and DNR-resistant K562 cells **(b)**. The cells were treated with Pt NPs, DNR or DNR@Pt NPs at the indicated concentrations and assayed after 48 h. Based on the mass fraction of Pt NPs and DNR in DNR@Pt NPs of76% and 24%, respectively, in the 10 μg mL^{-1} DNR@Pt NPs group, for example, the concentrations of Pt NPs and DNR were 7.6 μg mL^{-1} and 2.4 μg mL^{-1}, respectively. The bars in the graphs represent the results from three independent experiments. Data = Mean +/- Standard deviation (n=3).

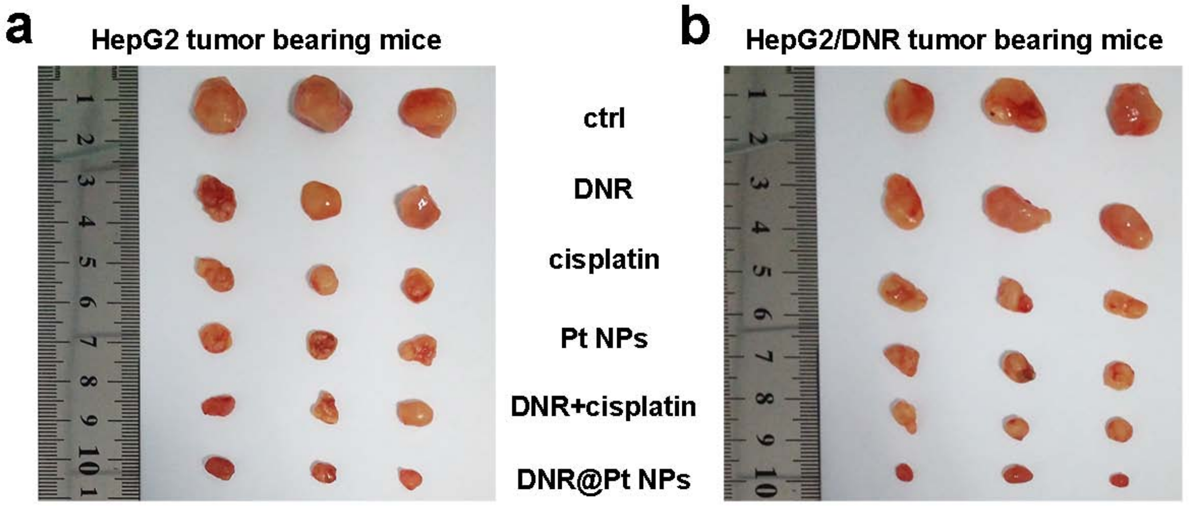

Supplementary Figure 19. (a) DNR-sensitive HepG2 and **(b)** DNR-resistant HepG2 bearing nude mice were treated with PBS (ctrl), 0.1 mg kg^{-1} DNR, 1 mg kg^{-1} cisplatin, 1 mg kg^{-1} Pt NPs, 0.1 mg kg^{-1} DNR combined with 1 mg kg^{-1} cisplatin or 1 mg kg^{-1} DNR@Pt NPs. The tumors were excised and photographed.

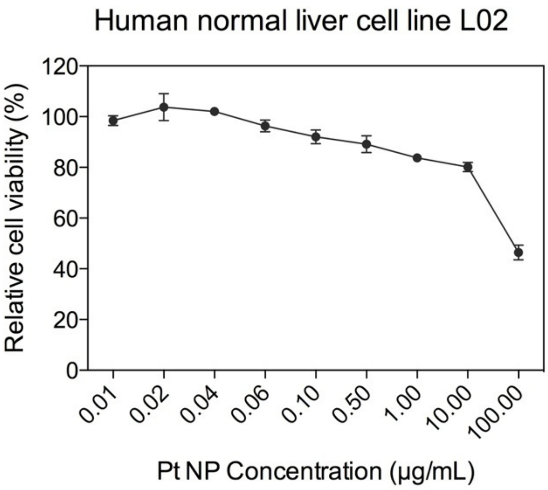

Supplementary Figure 20. Cell Counting Kit (CCK-8) assay for cell viability in the human normal liver cell line L02. The cells were treated with the indicated concentrations of Pt NPs and assayed after 48 h. The data points represent the results from three independent experiments. The IC_{50} of Pt NPs was calculated to be 98.51 μg mL^{-1}. Data = Mean +/- Standard deviation (n=3).

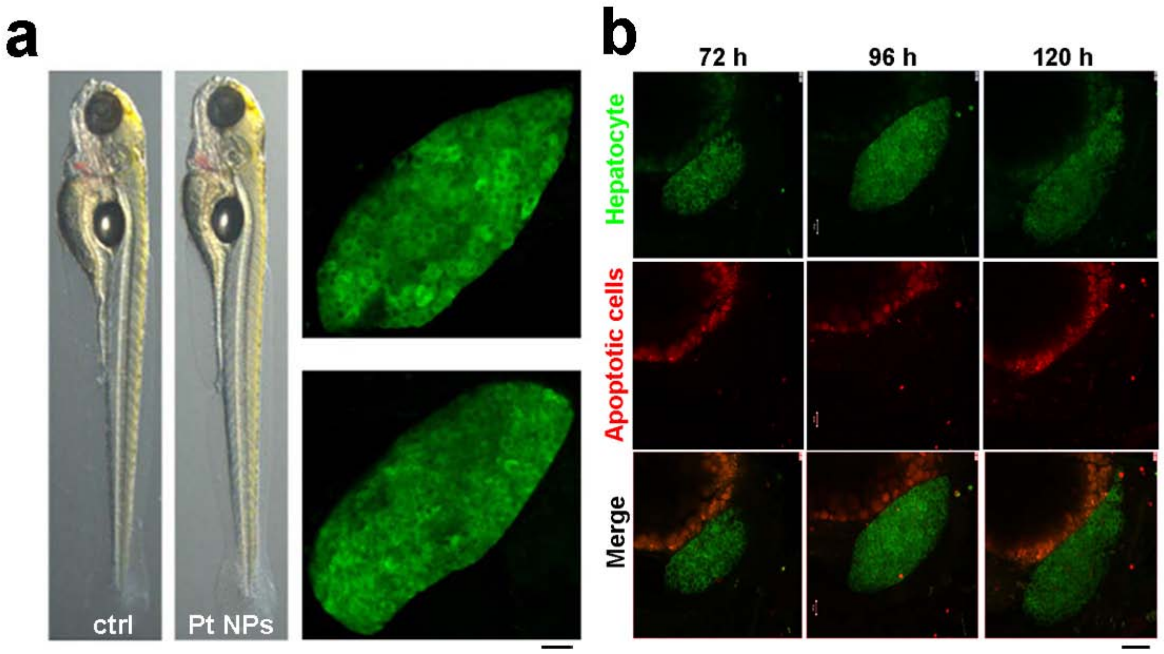

Supplementary Figure 21. (a) Representative micrographs of zebrafish embryos that were exposed to 1 mg L^{-1} Pt NPs for 5 days. The GFP-hepatocyte (green) transgenic zebrafish livers showed no change in size after the 1 mg L^{-1} Pt NP treatment. Scale bar = 30 μm. **(b)** After 1 mg L^{-1} Pt NP treatment for 72-120 h, TUNEL staining was performed to detect the apoptotic cells (red) in GFP transgenic zebrafish embryos; the tissue next to the green liver is red natural yolk. Scale bar = 50 μm.

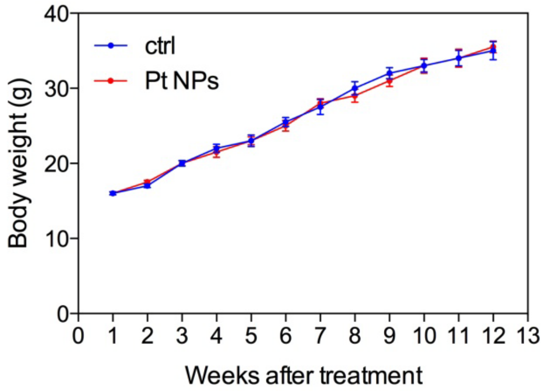

Supplementary Figure 22. Body weights of mice treated with Pt NPs (Red) or PBS vehicle control (Blue) for 12 weeks. The mice were treated with 5 mg kg^{-1} Pt NPs by intravenous injection once per week. Data = Mean +/- Standard deviation (n=5).

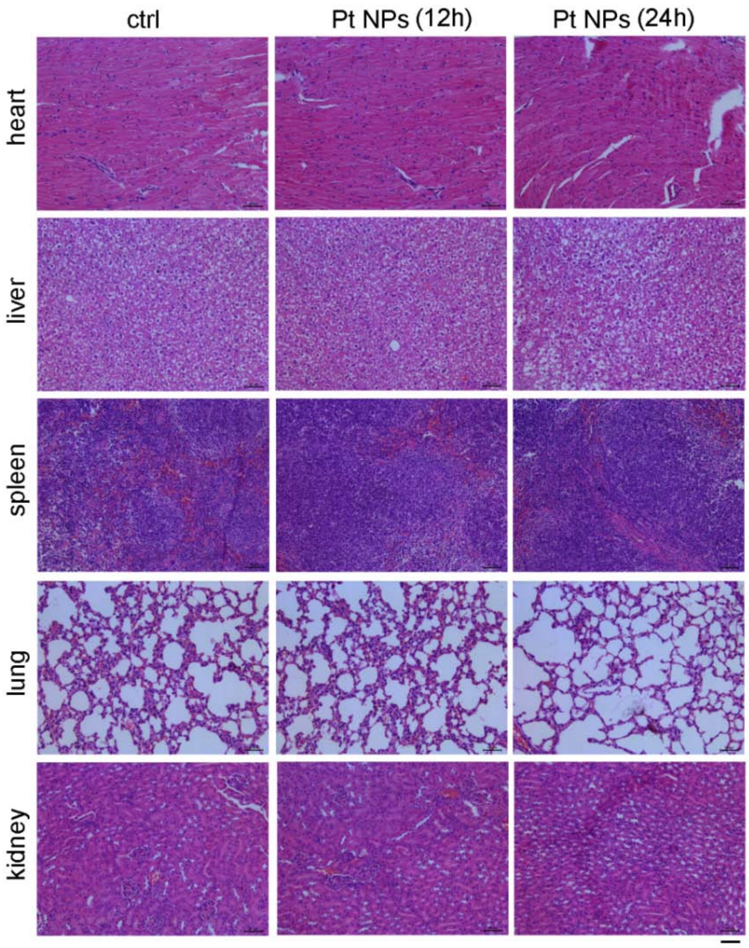

Supplementary Figure 23. Histological analysis of C57BL/6 mouse heart, liver, spleen, lung, and kidney after 1 mg kg^{-1} Pt NPs treatment for 12 and 24 h. Representative images from 5 mice in each group. Scale bar = 50 μm.

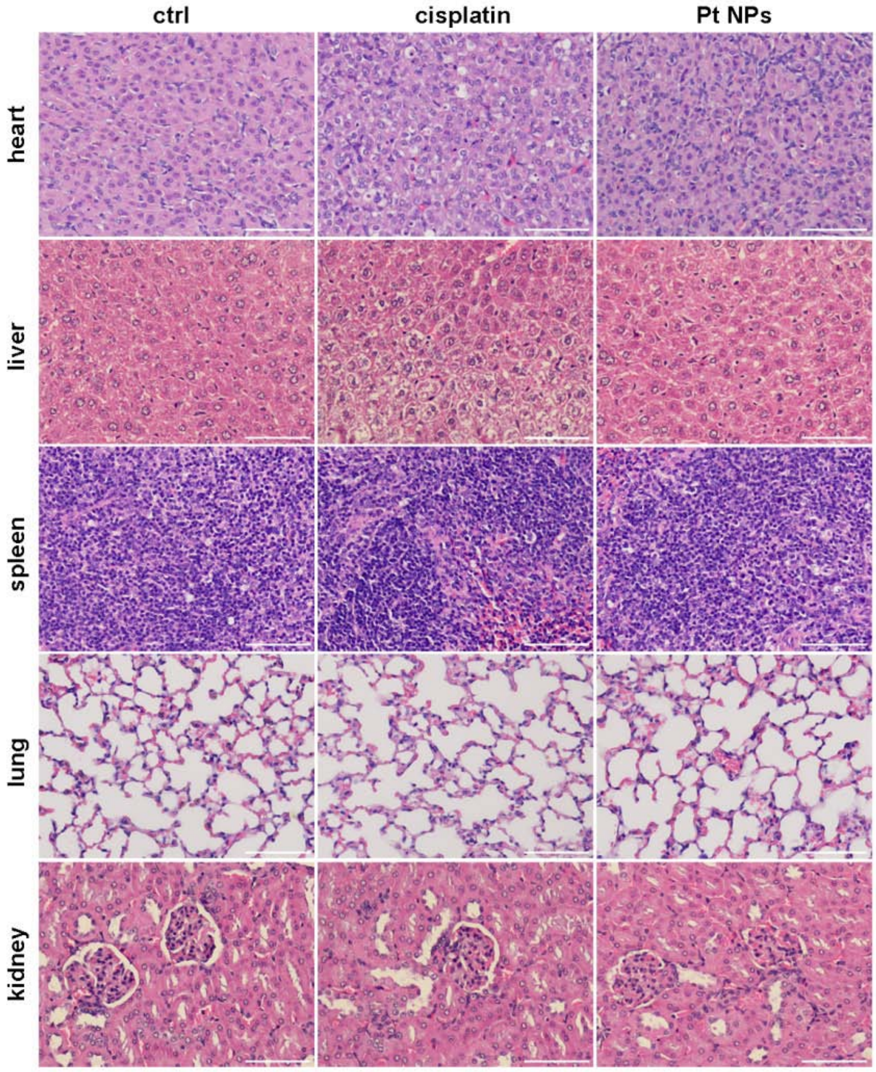

Supplementary Figure 24. Histological analysis of C57BL/6 mouse heart, liver, spleen, lung, and kidney after 0.1 mg kg^{-1} cisplatin or 1 mg kg^{-1} Pt NPs treatments for a total of 14 days (treatment every 2 days). Representative images from 5 mice in each group. Scale bar = 100 μm.

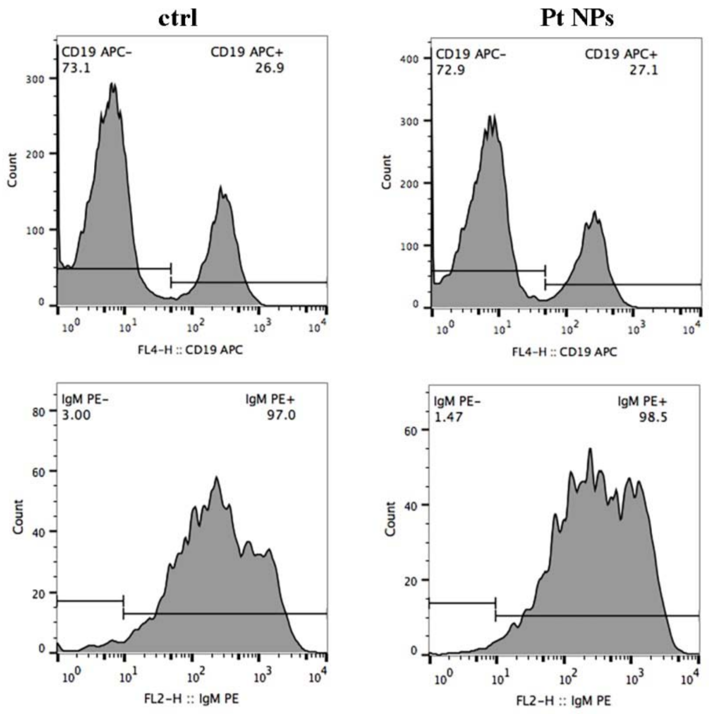

Supplementary Figure 25. C57BL/6 mice were treated with 1 mg kg^{-1} Pt NPs7 times over 72 h. Lymphocytes were isolated and subjected to flow cytometric analysis for CD19^{+}/IgM^{+} B cells.

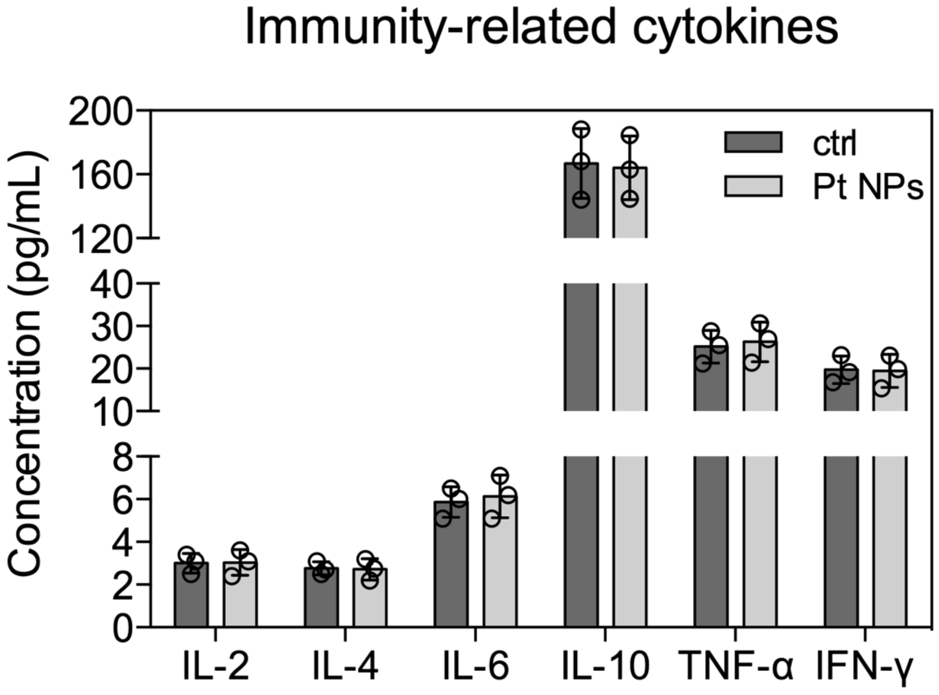

Supplementary Figure 26. C57BL/6 mice were treated with PBS or 1 mg kg^{-1} Pt NPs (for 72 h). The plasma was isolated after 72 h and subjected to flow cytometric analysis for innate immunity-related cytokines using mouse Th1/Th2/Th17 cytokine kit, including IL-2, IL-4, IL-6, IL-10, TNF-α and IFN-γ. Data = Mean +/- Standard deviation (n=3).

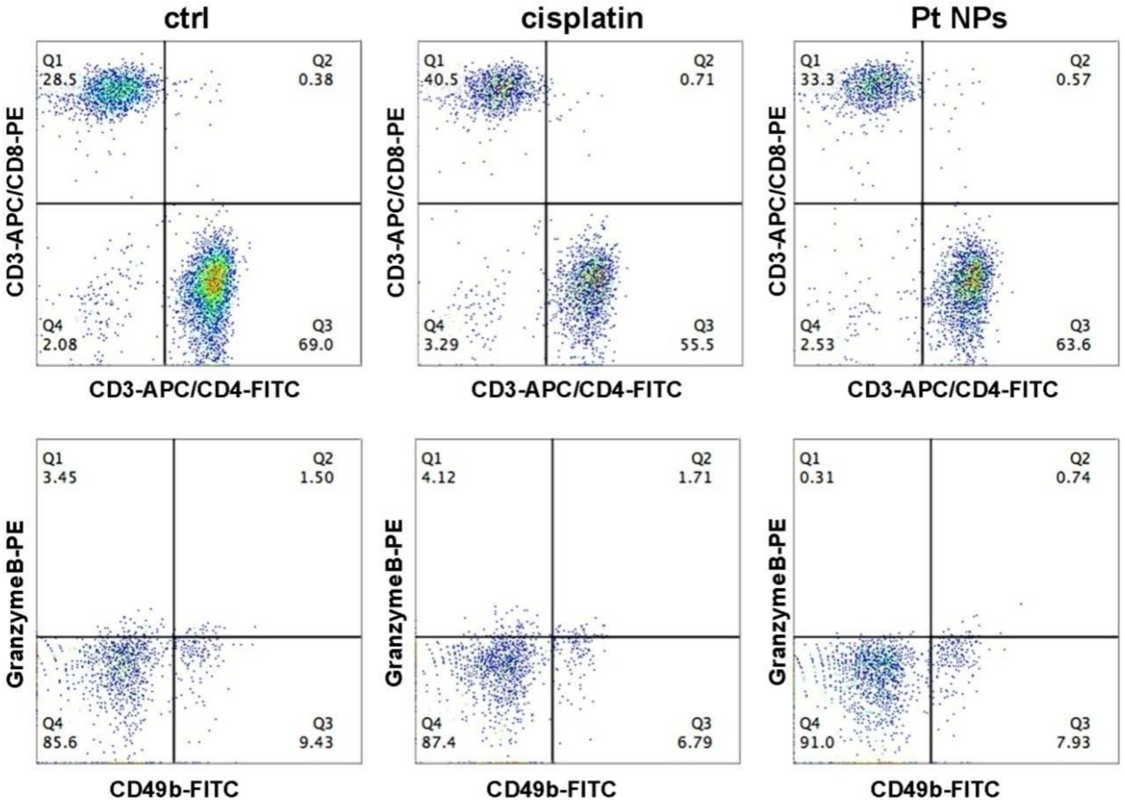

Supplementary Figure 27. C57BL/6 mice were treated with 0.1 mg kg^{-1} cisplatin or 1 mg kg^{-1} Pt NPs 7 times over 14 days. Lymphocytes were isolated and submitted to flow cytometric analysis for CD^{3+}/CD^{4+} T cells, CD^{3+}/CD^{8+} T cells and $CD49b^{+}$/$GranzymeB^{+}$ NK cells.

Supplementary Table 1. Cisplatin chemotherapy in cancer patients

	Number of patients	Treatment
Total	100 (person)	Cisplatin (mg m^{-2})
Gender		
Male	57	90
Female	43	90
Age at chemotherapy (years)		
≤60	22	90
>60	78	90
Sample		
Blood	100	
Urine	53	
Faeces	20	
Serum	8	0

Supplementary Table 2. Cisplatin chemotherapy in cancer patients after surgery

	Number of patients	Treatment
Total	10	Cisplatin (mg m^{-2})
Gender		
Male	6	90
Female	4	90
Age at chemotherapy (years)		
≤60	8	90
>60	2	90
Sample		
Tumor specimen	10	

Supplementary Table 3. DNR IC_{50} values and resistance index

Treatment	IC_{50} values		
	Pt NPs	DNR	DNR@Pt NPs
DNR-sensitive K562 cells	3.6642 μg mL^{-1}	0.0049 μg mL^{-1}	0.0144 μg mL^{-1}
DNR-resistant K562 cells	8.6580 μg mL^{-1}	0.0611 μg mL^{-1}	0.1150 μg mL^{-1}
Resistance Index	2.363	12.469	7.986

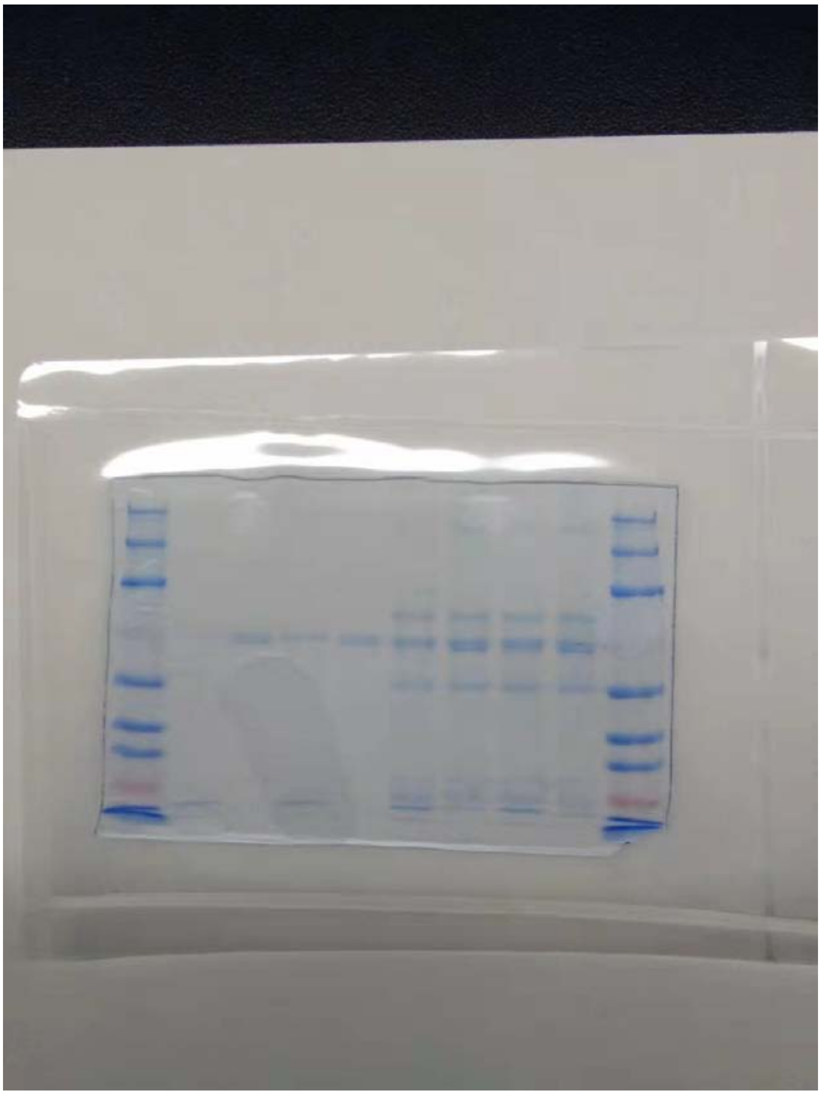

Supplementary Information. Uncropped image of data in supplementary figure 13.

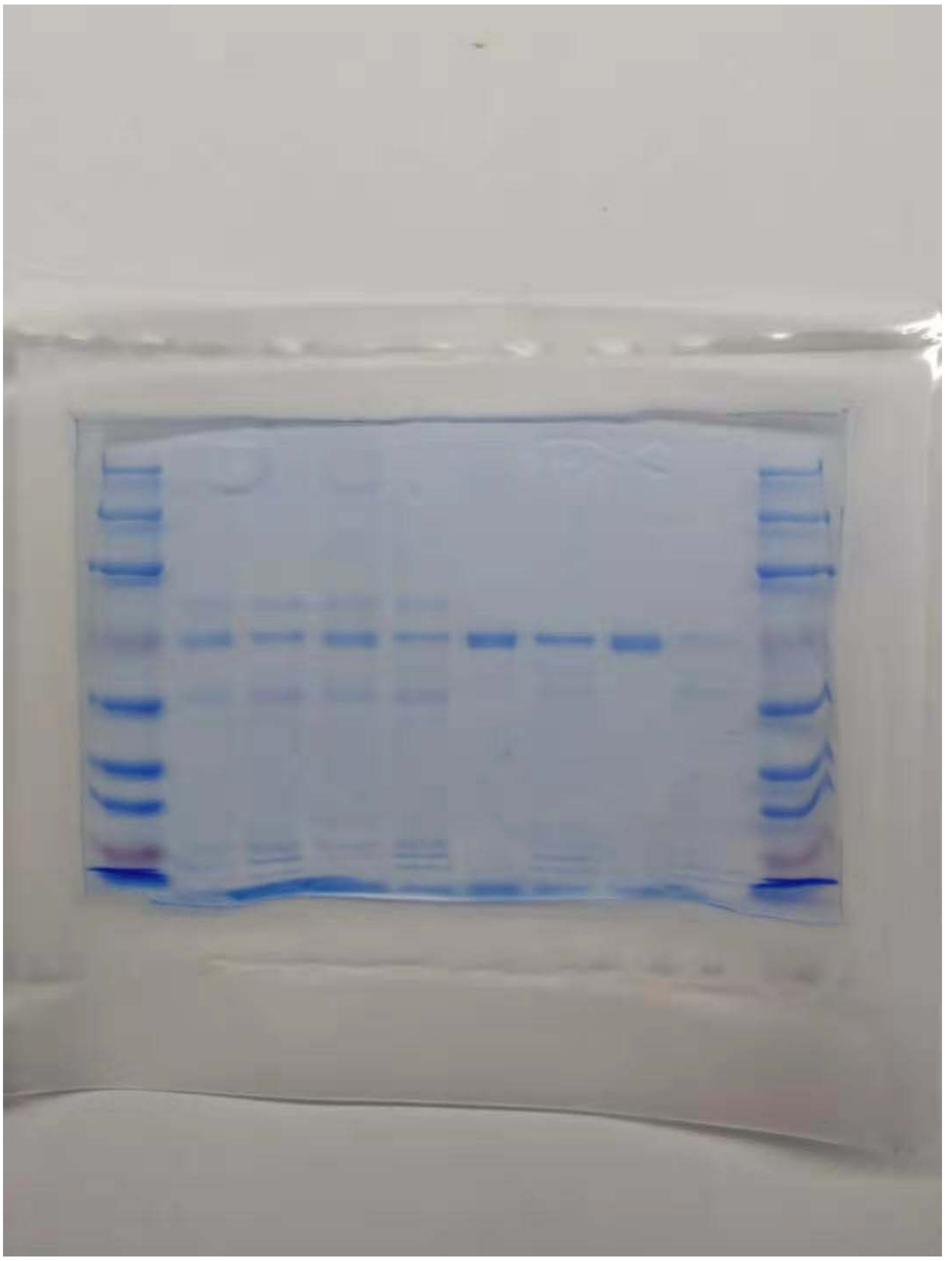

Supplementary Information. Uncropped image of data in supplementary figure 14.
