# Supplementary material for: Blood-triggered generation of platinum nanoparticle functions as an anti-cancer agent
Source: Nat Commun. 2020 Jan 28;11:567. doi: 10.1038/s41467-019-14131-z (PMC6987201; doi:10.1038/s41467-019-14131-z)
Supplement: Supplementary file 2 — Reporting Summary [file 41467_2019_14131_MOESM2_ESM.pdf]

nature research

Corresponding author(s): Gen Zhang

Last updated by author(s): Dec 17, 2019

Reporting Summary

Nature Research wishes to improve the reproducibility of the work that we publish. This form provides structure for consistency and transparency in reporting. For further information on Nature Research policies, see Authors & Referees and the Editorial Policy Checklist .

Statistics

For all statistical analyses, confirm that the following items are present in the figure legend, table legend, main text, or Methods section.

n/a	Confirmed	
☐	☒	The exact sample size (*n*) for each experimental group/condition, given as a discrete number and unit of measurement
☐	☒	A statement on whether measurements were taken from distinct samples or whether the same sample was measured repeatedly
☐	☒	The statistical test(s) used AND whether they are one- or two-sided<br>*Only common tests should be described solely by name; describe more complex techniques in the Methods section.*
☒	☐	A description of all covariates tested
☒	☐	A description of any assumptions or corrections, such as tests of normality and adjustment for multiple comparisons
☐	☒	A full description of the statistical parameters including central tendency (e.g. means) or other basic estimates (e.g. regression coefficient) AND variation (e.g. standard deviation) or associated estimates of uncertainty (e.g. confidence intervals)
☒	☐	For null hypothesis testing, the test statistic (e.g. *F*, *t*, *r*) with confidence intervals, effect sizes, degrees of freedom and *P* value noted<br>*Give P values as exact values whenever suitable.*
☒	☐	For Bayesian analysis, information on the choice of priors and Markov chain Monte Carlo settings
☒	☐	For hierarchical and complex designs, identification of the appropriate level for tests and full reporting of outcomes
☒	☐	Estimates of effect sizes (e.g. Cohen's *d*, Pearson's *r*), indicating how they were calculated

Our web collection on statistics for biologists contains articles on many of the points above.

Software and code

Policy information about availability of computer code

Data collection	*Provide a description of all commercial, open source and custom code used to collect the data in this study, specifying the version used OR state that no software was used.*
Data analysis	*Provide a description of all commercial, open source and custom code used to analyse the data in this study, specifying the version used OR state that no software was used.*

For manuscripts utilizing custom algorithms or software that are central to the research but not yet described in published literature, software must be made available to editors/reviewers. We strongly encourage code deposition in a community repository (e.g. GitHub). See the Nature Research guidelines for submitting code & software for further information.

Data

Policy information about availability of data

All manuscripts must include a data availability statement. This statement should provide the following information, where applicable:
- Accession codes, unique identifiers, or web links for publicly available datasets
- A list of figures that have associated raw data
- A description of any restrictions on data availability

A list of figures that have associated raw data

Field-specific reporting

Please select the one below that is the best fit for your research. If you are not sure, read the appropriate sections before making your selection.

☒ Life sciences ☐ Behavioural & social sciences ☐ Ecological, evolutionary & environmental sciences

Life sciences study design

All studies must disclose on these points even when the disclosure is negative.

Sample size	samples sizes were chosen based on previous literature and in order to allow for statistical analysis
Data exclusions	no data were excluded from the analyses
Replication	all attempts at replication were successful
Randomization	the samples/participants were randomly allocated into experimental groups
Blinding	the investigators were blinded to group allocation during data collection and analysis

Reporting for specific materials, systems and methods

We require information from authors about some types of materials, experimental systems and methods used in many studies. Here, indicate whether each material, system or method listed is relevant to your study. If you are not sure if a list item applies to your research, read the appropriate section before selecting a response.

Materials & experimental systems

n/a	Involved in the study	
☐	☒	Antibodies
☐	☒	Eukaryotic cell lines
☒	☐	Palaeontology
☐	☒	Animals and other organisms
☐	☒	Human research participants
☒	☐	Clinical data

Methods

n/a	Involved in the study	
☒	☐	ChIP-seq
☐	☒	Flow cytometry
☒	☐	MRI-based neuroimaging

Antibodies

Antibodies used	caspase-3(CST, #9664), caspase-8(CST, #9496), caspase-9(CST, #7237). all informations about the antibodies were provided in Methods section.
Validation	these data was provided in the main manuscript.

Eukaryotic cell lines

Policy information about cell lines

Cell line source(s)	K562, HepG2, L02
Authentication	All cell lines were purchased from ATCC (American Type Culture Collection)
Mycoplasma contamination	all cell lines tested negative for mycoplasma contamination
Commonly misidentified lines (See ICLAC register)	No misidentified line was used in this study

Animals and other organisms

Policy information about studies involving animals; ARRIVE guidelines recommended for reporting animal research

Laboratory animals	BALB/C nude mice, C57BL/6 mice, male:female=50%:50%
Wild animals	the study did not involved wild animals
Field-collected samples	the study did not involved samples collected from the field
Ethics oversight	Animal Research Ethics Board of Nanjing Medical University

Note that full information on the approval of the study protocol must also be provided in the manuscript.

Human research participants

Policy information about studies involving human research participants

Population characteristics	males 63, female 47. All the other informations about the populations were supplied in the Supplementary Tables.
Recruitment	110
Ethics oversight	Nanjing Medical University Management Committee and Ethics Committee

Note that full information on the approval of the study protocol must also be provided in the manuscript.

Flow Cytometry

Plots

Confirm that:

- [x] The axis labels state the marker and fluorochrome used (e.g. CD4-FITC).
- [x] The axis scales are clearly visible. Include numbers along axes only for bottom left plot of group (a 'group' is an analysis of identical markers).
- [x] All plots are contour plots with outliers or pseudocolor plots.
- [x] A numerical value for number of cells or percentage (with statistics) is provided.

Methodology

Sample preparation	mouse blood cells
Instrument	BD FACS Calibur
Software	BD FACS Calibur
Cell population abundance	10000 cells in gate
Gating strategy	based on the values of FSC/SSC

- [ ] Tick this box to confirm that a figure exemplifying the gating strategy is provided in the Supplementary Information.
